# A new dynamic correlation algorithm reveals novel functional aspects in single cell and bulk RNA-seq data

**DOI:** 10.1371/journal.pcbi.1006391

**Published:** 2018-08-06

**Authors:** Tianwei Yu

**Affiliations:** Department of Biostatistics and Bioinformatics, Emory University, Atlanta, GA, United States of America; Ottawa University, CANADA

## Abstract

Dynamic correlations are pervasive in high-throughput data. Large numbers of gene pairs can change their correlation patterns in response to observed/unobserved changes in physiological states. Finding changes in correlation patterns can reveal important regulatory mechanisms. Currently there is no method that can effectively detect global dynamic correlation patterns in a dataset. Given the challenging nature of the problem, the currently available methods use genes as surrogate measurements of physiological states, which cannot faithfully represent true underlying biological signals. In this study we develop a new method that directly identifies strong latent dynamic correlation signals from the data matrix, named DCA: Dynamic Correlation Analysis. At the center of the method is a new metric for the identification of pairs of variables that are highly likely to be dynamically correlated, without knowing the underlying physiological states that govern the dynamic correlation. We validate the performance of the method with extensive simulations. We applied the method to three real datasets: a single cell RNA-seq dataset, a bulk RNA-seq dataset, and a microarray gene expression dataset. In all three datasets, the method reveals novel latent factors with clear biological meaning, bringing new insights into the data.

## Author summary

Dynamic correlation is an important area in expression data. However it hasn’t received much attention because of the lack of effective methods that can unravel the complex relationship. Here we describe a new method that represents a substantial improvement over existing approaches. It achieves the goal of efficiently finding patterns of dynamic correlation in RNA-seq data, as well as detecting biological functions associated with the dynamic correlation patterns. Unlike traditional methods that focus on first-order structures, linear or nonlinear, our method finds second-order patterns that bring insights into the regulations of the complex system. Some of the interesting discoveries by the new method, such as immunological functions of some intestinal epithelial cells, are validated by recent biological publications.

## Introduction

The biological system involves tens of thousands of genes/proteins that are tightly regulated in a complex network [1–3]. Interactions and regulations in the network are highly dynamic. They change substantially in different cell types, developmental stages, or in response to environmental conditions [4]. Gene expression and similar types of data, such as proteomics and metabolomics data, represent outcomes of the dynamic regulatory network. Changes in the underlying regulation patterns can often result in changes in correlation between genes.

In many gene expression profiling datasets, the cellular states or sub-classes are not observed directly. Thus dynamic correlation needs to be inferred from the data. Once successfully extracted from the data, the dynamic correlation patterns can in-turn help deduce hidden cellular states and sub-classes. The most common dynamic correlation takes following form: the correlation between a pair of genes ***g***_***i***_ and ***g***_***j***_ is reliant on the value of an unobserved variable *Z*, i.e. *cor(****g***_***i***_**, *g***_***j***_*) = f(Z)*, where *f()* is an unspecified monotone function. *Z* can be the activity of a specific regulator in the system, or it can be the reflection of cellular states resulting from the collective activities of multiple regulators. Because gene expression is tightly controlled in the cell, the same *Z* variable can govern the dynamic correlation of many gene pairs.

Given the complexity of cellular regulations, systematically studying dynamic correlation is challenging. First, as the biological system is organized in a modular manner [5], there could be multiple *Z* variables that govern the dynamic correlation of different groups of genes. Secondly, the underlying cellular states may not manifest into biological/clinical observations, making most of the *Z* variables unobservable. Hence the major interest is to find the unobserved *Z* variables. To this end, Li has developed the Liquid Association (LA) approach, which uses genes as proxy measurements of the unobserved *Z* variables [6, 7]. The method scans through all possible gene triplets to find potential dynamic correlations. Similar approaches that utilize genes as mediators [8, 9], integrative analysis utilizing Liquid Association [10, 11], as well as statistical theory of Liquid Association [12] were later developed.

Although using genes as surrogate measurements of the *Z* variables can reveal some important local regulatory mechanisms, a more global approach to dynamic correlation could discover critical regulation mechanisms that penetrate multiple biological processes, or help identify hidden sub-groups in the samples. To this end, using the original LA or similar approaches is not effective due to the following reasons. First, scanning through all possible gene triplets is computationally intensive. Second, a genome-scale scan yields large numbers of significant gene triplets, causing difficulties in the interpretation. Given the LA score is calculated in a symmetric manner among the three genes involved, discerning which gene reflects cellular states could be tricky. Third and the most important, measurements in the genes may not be good indicators of the true underlying cellular states.

In this study, our purpose is to find latent signals that govern the dynamic correlation of a large number of gene pairs. The key differences between our approach and screening by Liquid Association are: (1) We do not assume the signals that control the dynamic correlation of gene pairs are contained in any gene; (2) We are only interested in finding the dominating dynamic correlation signals that impact large numbers of gene pairs, but not local signals that govern only a small number of gene pairs. (3) Compared to screening all gene triplets by Liquid Association, the method is magnitudes faster.

To develop such a method, the biggest difficulty is we do not know *a priori* which gene pairs are dynamically correlated. To solve this problem, we design a new metric, named Liquid Association Coefficient (LAC), to effectively and efficiently screen all gene pairs for potential dynamic correlations. From gene pairs that are most likely to be dynamically correlated, we provide a simple and straight-forward solution for quickly finding the latent dynamic correlation signals. The procedure is named DCA: Dynamic Correlation Analysis. We refer to the latent signals found by DCA as Dynamic Components (DCs).

We demonstrate the performance of the method using extensive simulations. In real biological datasets, the method can identify latent signals that are biologically meaningful and not found by existing methods. In a single cell RNA-seq dataset, DCA was able to separate more cell types and shed light on the biological functions that drove the separation. In the Cancer Genome Atlas (TCGA) breast cancer (BRCA) dataset, DCA found new interesting subgroups in the subjects that are related to patient survival outcome. In a merged cell cycle dataset, the method recovered signals pertaining to the original experimental grouping, as well as biological processes that differentiate the experiments, shedding lights on the side-effects of the data-generation process.

## Results

### Behavior of the Liquid Association Coefficient (LAC)

In this study, a new metric was defined to rank all pairs of variables in the data matrix. The purpose of the LAC was to help identify gene pairs that were most likely to have the relationship of dynamic correlation, without knowing the underlying physiological states that govern the dynamic correlation. Gene pairs with such relations should receive high LAC score, while other gene pairs, either independent or correlated, should receive low scores.

**Fig 1:**
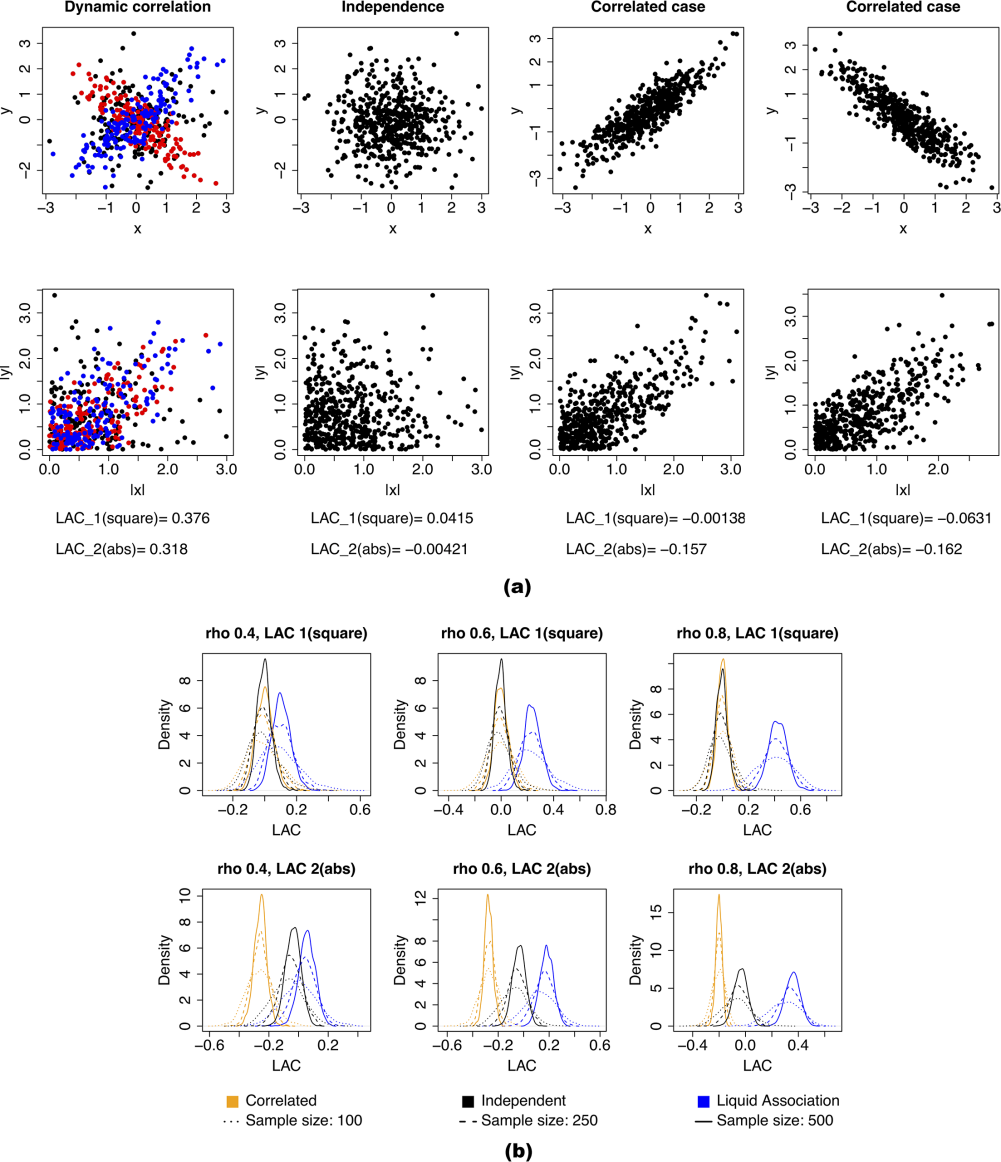
The liquid association coefficient (LAC). **(a) Illustration of LAC using examples.** Left column: dynamic correlation with an unknown conditioning factor. When the factor is low, *x* and *y* are negatively correlated; when the factor is high, *x* and *y* are positively correlated. Second left column: independent case. Right two columns: correlated case. In all the cases, the marginal distribution of *X* and *Y* are standard normal. **(b) Empirical distributions of LAC score under conditions of dynamic correlation, simple correlation, or independence.** The densities are based on 1000 simulations. In the dynamic correlation cases, one-third of the data points follow a bivariate normal distribution with mean $\left(\begin{array}{c} 0\\ 0 \end{array}\right)$ and variance-covariance matrix $\left(\begin{array}{cc} 1 & \rho \\ \rho & 1 \end{array}\right)$, one-third follow a bivariate normal distribution with mean $\left(\begin{array}{c} 0\\ 0 \end{array}\right)$ and variance-covariance matrix $\left(\begin{array}{cc} 1 & -\rho \\ -\rho & 1 \end{array}\right)$, and another one-third follow independent standard normal distributions. In the correlated case, all data points follow a bivariate normal distribution with mean $\left(\begin{array}{c} 0\\ 0 \end{array}\right)$ and variance-covariance matrix $\left(\begin{array}{cc} 1 & \rho \\ \rho & 1 \end{array}\right)$.

The LAC requires all variables to have mean zero and standard deviation 1. As illustrated in Fig 1A, if both variables X and Y followed the standard normal distribution marginally, and one-third of the (X,Y) pairs were positively correlated, one-third of the (X,Y) pairs were negatively correlated, and another one-third uncorrelated, then the absolute values would be positively correlated, and the *LAC* tended to be large (Fig 1A, left column). On the other hand, when X and Y were truly independent or simply correlated, the *LAC* tended to be small.

We further conducted a simulation study to examine the empirical distribution of LAC under different circumstances. As illustrated in Fig 1B, when the two variables were dynamically correlated, the distribution of the LAC score was centered at a positive value (Fig 1B, blue curves). The higher the correlation level, the higher the mean (Fig 1B, left to right panels). The higher the sample size, the less the spread (Fig 1B, different line types). At the same time, in the independent and correlated cases, the LAC scores were centered around zero if the first definition of *LAC* is used. Using the second definition, the *LAC* was still centered around zero in the independent case, and the center was negative in the correlated case (Fig 1B, lower panels). Intuitively for the correlated case, when taking the absolute value, the range of |*x*| became smaller than *x* itself, while the spread of data points around the trendline stayed the same. This meant the correlation between the absolute values tended to become smaller than the original, resulting in a negative LAC score.

### Simulation study

We conducted an extensive simulation study to evaluate the method’s capability to recover latent dynamic correlation signals. Each simulated dataset was made of multiple modules, each of which was regulated by a single underlying dynamic correlation factor. To simulate a module of genes that have dynamic correlation conditioned on the same factor, we first simulated the latent factor z by sampling the standard normal distribution. For the conditional correlation pattern, we simulated three different fashions separately: (1) *E*(*XY*|*z*) = (Φ^−1^(*z*)−0.5) × 2; (2) Truncate z at -3.2 and 3.2, and then $E(XY|z)=sign(z)\times \sqrt{\left|z/3.2\right|}$; (3) Truncate z at -3.2 and 3.2, and then $E(XY|z)=sign(z)\times \sqrt[{4}]{\left|z/3.2\right|}$.

We then generated 10 pairs of seed vectors (***x*, *y***) such that each random variable followed the standard normal distribution marginally, and between a pair of X and Y, their correlation was dependent on z. The details of generating an ***(x*, *y)*** pair were as follows:

1. For each *z* value, we found the conditional correlation value *ρ*_*z*_ = *E*(*XY*|*z*) between *X* and *Y* based on the three scenarios above, for example, *ρ*_*z*_ = *E*(*XY*|*z*) = (Φ^−1^(*z*)−0.5) × 2;
2. We sampled one data point from the two-dimensional Gaussian distribution with mean vector $\left(\begin{array}{c} 0\\ 0 \end{array}\right)$ and variance-covariance matrix $\left(\begin{array}{cc} 1 & \rho_{z}\\ \rho_{z} & 1 \end{array}\right)$;
3. We iterated steps (1) and (2) through all *N* values of the ***z*** vector, to obtain *N* pairs of *(x*, *y)*. Together they made the two vectors that were dynamically correlated conditioned on *Z*.

For each ***z*** vector, after repeating the above process and generating 10 pairs of such seed vectors, we used the following procedure to generate the observed expression vectors:

1. We randomly selected one pair from the seed vector pairs;
2. We added Gaussian noise to the selected seed vector to generate one pair of simulated genes;
3. We repeated steps (1) and (2) until the desired number of simulated genes were generated.

In each simulation dataset, multiple ***z*** vectors were generated. From each of the ***z*** vectors, a group of genes that were dynamically correlated conditioned on the ***z*** vector were generated. In addition, noise genes were generated by sampling from the standard normal distribution. The number of noise genes was equal to the total number of genes involved in dynamic correlation modules.

In order to mimic the situation where the data are highly skewed and zero-inflated as in RNA-seq data, we also conducted another set of simulations. First, we simulated data with normal marginal distribution using the procedure above. Then for each simulated gene, we randomly drew one gene from the TCGA BRCA dataset that had less than 75% zero values, and matched the quantiles of the simulated gene to those of the real gene using the interpolating quantile normalization procedure described in [13]. This approach forced each simulated gene to have the same marginal distribution as a real gene.

After generating 50 datasets in each simulation setting, we compared DCA with six other methods. The first was screening by Liquid Association. Conceptually, this would involve computing the LA score for all possible gene triplets, and then selecting the top LA scouting genes that were involved in the highest numbers of triplets with high-LA scores. However, the heavy computational cost of screening through all $\left(\begin{array}{c} p\\ 3 \end{array}\right)$ triplets made it impractical to actually conduct the computation on all simulated datasets. Rather, the “LA screening” results were obtained by directly selecting the genes that had the highest absolute correlation with the true hidden factors, one gene for each factor. Given LA screening can only find signals that are in the genes, the simulation obtained the best possible results of LA screening, which showed the upper limit of how well LA screening could recover the dynamic correlation signal. But such results may not be attainable in actual computation. The other methods compared were dimension reduction methods: Principal Component Analysis (PCA), Independent Component Analysis (ICA), t-Distributed Stochastic Neighbor Embedding (t-SNE), kernel PCA with degree-two polynomial kernel, and kernel PCA with radial basis function kernel.

For comparison, assuming there were K true latent factors, each method was allowed to find the top K+2 factors, except for LA screening, which found K factors as discussed above. Among the found factors, K of them were paired with the latent factors, by sequentially seeking the highest absolute correlation coefficient between any found factor and latent factor. We then calculated the absolute correlation coefficient between the hidden factors and their paired true hidden factors, and found the average absolute correlation for each simulation setting as the indication of how well the latent factors were recovered.

**Fig 2:**
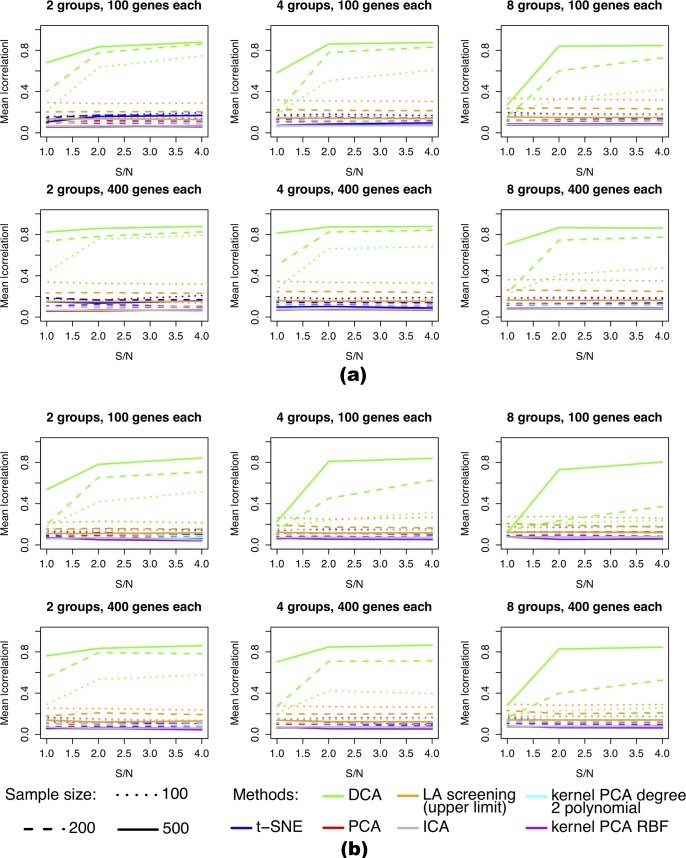
Simulation result–the average absolute Spearman correlation between latent factors and their corresponding found factors. (a) The marginal distributions of gene expression levels were normal. (b) The marginal distributions of gene expression levels mimicked real RNA-seq data. Row sub-plots: number of genes in each module; Columns subplots: the number of modules; Line type: sample size; line color: method used for latent factor recovery. Given the heavy computational cost, the “LA screening (upper limit)” results were obtained by directly selecting the genes that have the highest absolute correlation with the hidden factors, meaning the values plotted are the best possible, but may not be attainable in actual computation.

In setup 1, when the marginal distribution of gene expression was normal, DCA recovered the latent signals when signal to noise ration (S/N) and the sample size were moderate to high (Fig 2A). When the number of modules increased (Fig 2A, left to right), the capability to recover the latent factors decreased for lower sample sizes. At the same time, other methods failed to recover the latent factors. As the likelihood to generate spurious correlation was higher at smaller sample sizes, the dotted curves (low sample size) of LA screening and other methods were higher than the corresponding dashed and solid curves (higher sample sizes). However, this only reflected spurious correlations, rather than actual recovery of true signals.

The same trend held true for the datasets in which the marginal distribution of gene expression values mimicked the real data (Fig 2B). With the highly skewed and zero-inflated data, the faithful recovery of the hidden signals required more sample size and higher signal to noise ratio, compared to normally distributed data. Nevertheless, DCA was the only method that was capable of recovering the hidden signals.

Setup 2 was a weaker LA relationship than setup 1. As expected, the average absolute correlation was lower compared to setup 1 (S1 Fig). However the overall trend was the same–DCA recovered part of the latent variables, while other methods failed to recover the latent variables.

In setup 3, there were less extreme correlations between X and Y compared to setup 1. But at the same time, there were less low-correlation X-Y pairs. Overall the performance was similar to setup 1 (S2 Fig). In this setup, DCA performed better than setup 1 when sample size was small. It still recovered the latent variables at small sample sizes when the total number of modules were small (S2 Fig, left columns). Again the other methods failed to recover the latent variables. Overall, in all three setups, our method could faithfully recover the hidden dynamic correlation signal when the sample size and signal strength was sufficient.

### Real data analysis—single cell RNA-seq dataset of small intestinal epithelium cells

We used the single cell RNA seq data from the GSE92332 dataset [14]. The dataset contains measurements in mouse small intestinal epithelium cells under both normal condition and enteric pathogen treatments. For pattern detection we used the normal cells only. The data contains the measurement of 20108 genes measured in 1522 cells falling into nine types that were defined by known cell type-specific marker genes. For pattern detection using DCA, we removed genes with >25% zero counts. Given the sequencing depth, the remaining matrix contained 3041 genes.

**Fig 3:**
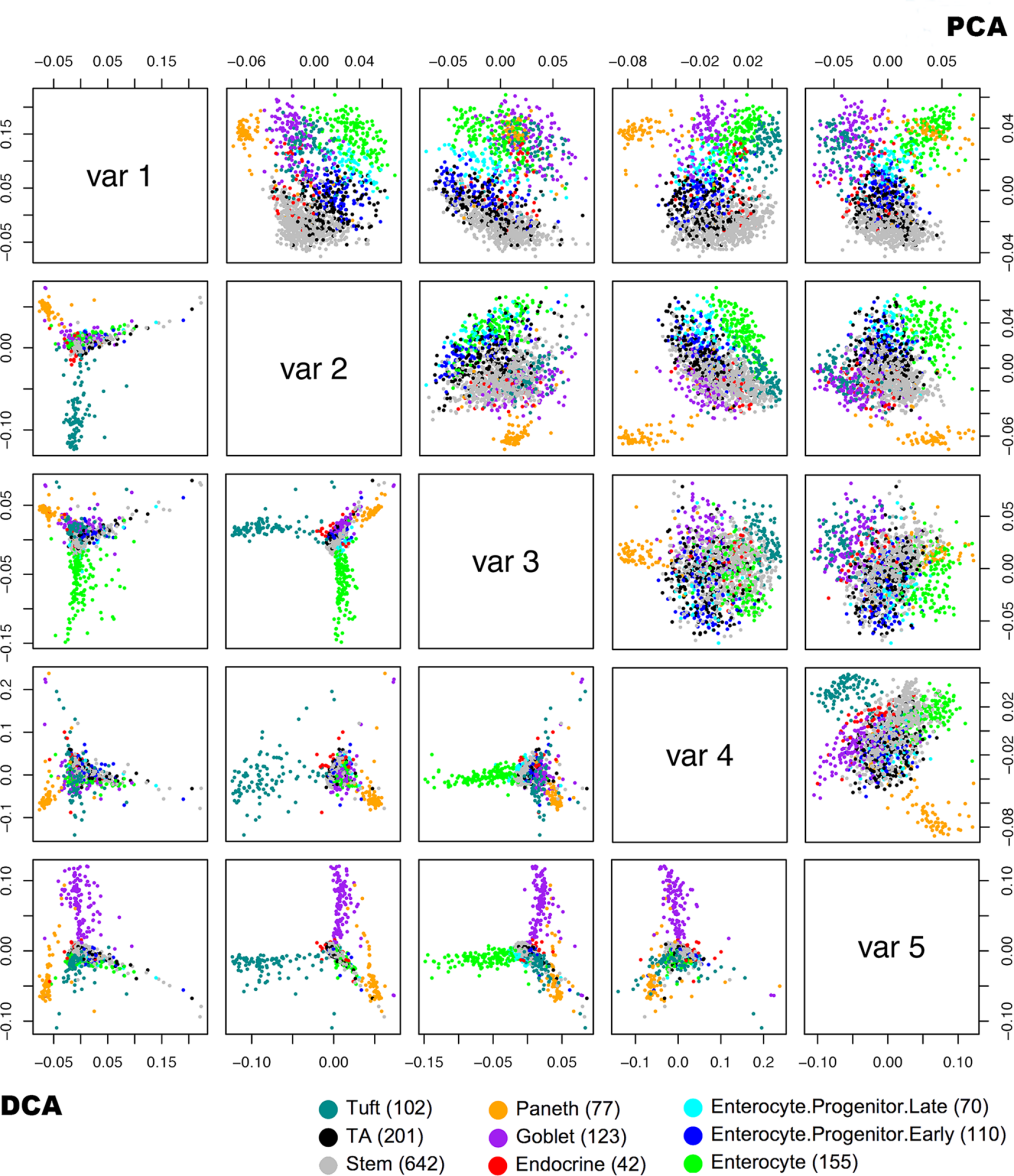
**Scatter plots of the first five DCs (lower left) and PCs (upper right) from the GSE92332 single cell RNA-seq data. The cells were colored using cell types based on known markers**.

We first examined the scores of the top latent factors (Fig 3). The score of each latent factor was a vector of 1522 values, corresponding to the 1522 cells. Every point in a subplot of Fig 3 represents a cell. As shown by the color of the points using the cell type information, the first 5 DCs clearly separated 4 types of the cells from the rest, and the separation was quite clear (Fig 3, lower-left sub-plots). As a comparison, the first 5 PCs only separated 2 cell types from the rest (Fig 3, upper-right sub-plots). Although there are some separations between the cell types when the points are colored by cell type, without the coloring, we would not be able to delineate the cell types clearly from the point patterns.

**Fig 4:**
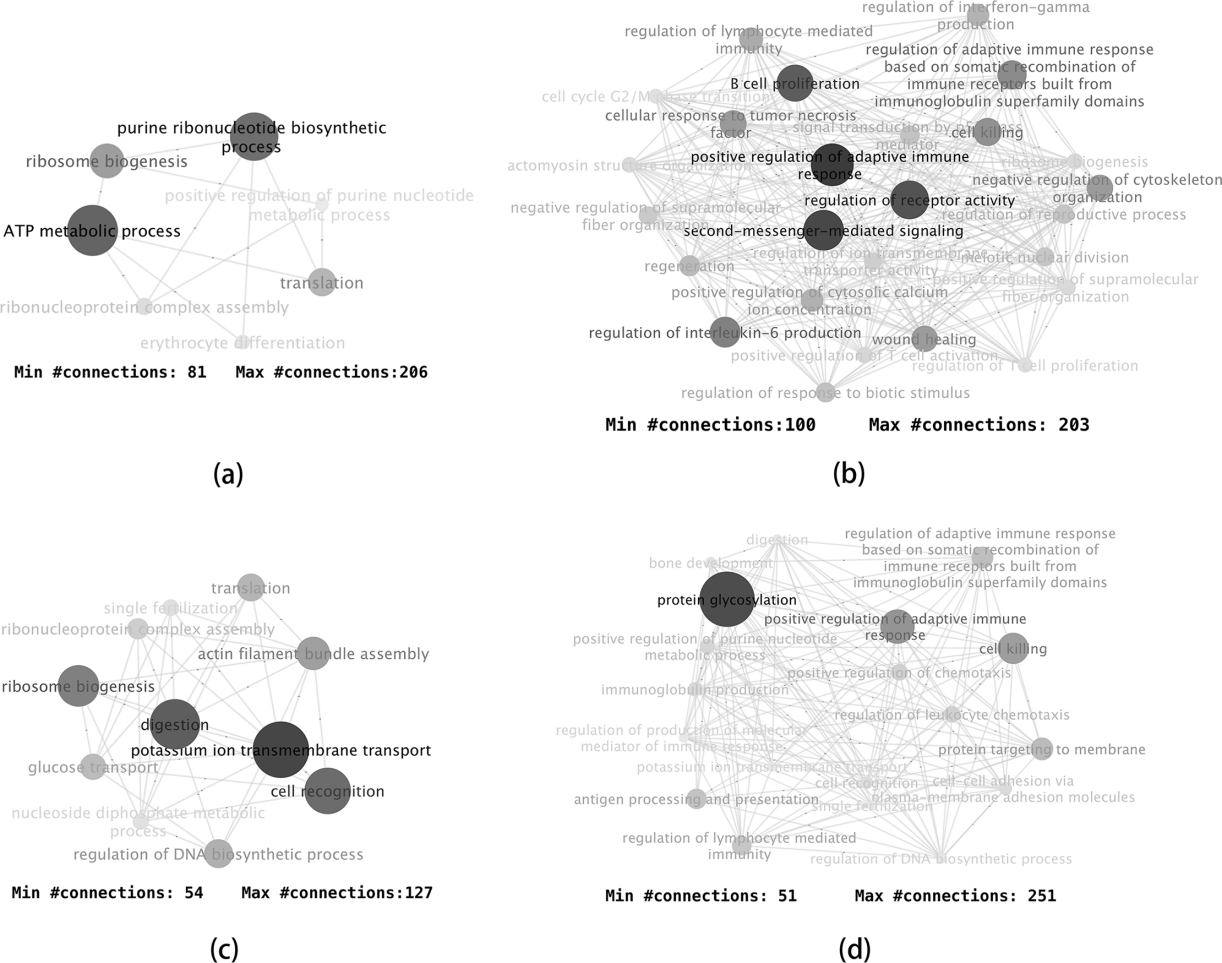
Major biological processes associated with the DCs. (a) DC1, (b) DC2, (c) DC3, and (d) DC5. Gene pairs were selected using fdr threshold of 0.01. Biological process pairs were selected using a p-value threshold of 0.001 and fold-change of 4. All were limited to biological processes with 50 or more connections, except for DC2, for which the limit was 100 due to the existence of excessive connections.

We next examined the biological processes whose differential correlation separated the cell types (Fig 4). DC1 mostly separated paneth cells from the rest. The function of paneth cells is mostly the secretion of anti-microbial proteins and peptides [15]. As shown in Fig 4A, the biological processes associated with DC1 were clearly concentrated in protein synthesis and energy production, which indicated protein/peptide biosynthesis was a critical functional aspect that separates the paneth cells from the rest.

DC2 mostly separated tuft cells from other cells. Tuft cells had long been considered a sensory cell. Only recently was tuft cell determined to be an important cell in innate immune response. Tuft cell secretes IL25 to stimulate the proliferation of innate lymphoid cells (ILC2s), and forms a feed-forward loop with ILC2s to generate type 2 immunity [16]. Our results showed many immune regulation processes and signaling processes were among the top biological processes associated with DC2 (Fig 4B). The results strongly agreed with the immunological function of tuft cells.

DC3 separated enterocytes from the other types of cells. Enterocytes are intestinal absorptive cells. The top biological processes included “digestion” and “potassium ion transmembrane transport” (Fig 4C), which includes the sodium-potassium pumps that are essential for the co-transport mechanism to absorb glucose and amino acids into the blood stream [17]. Some processes related to macromolecule biosynthesis were also among the highly connected.

DC5 separated goblet cells from the rest. Goblet cells secrete mucins, which are large glycoproteins, in order to protect the mucous membrane. Unsurprisingly, the major biological process that was associated with DC5 was protein glycosylation (Fig 4D). Interestingly, most other highly connected biological processes were immune-related functions. Some studies have started to confirm that the goblet cells actually have major immune functions [18], such as working as antigen retrievers [19]. The results here indicated a number of immune processes were activated at the transcription level.

From the pattern detection perspective, if the cell types were hidden, DCA clearly extracted more meaningful information to help differentiate the cell types, as well as points to important pathways that cause the distinction. In most real applications of dimension reduction, information such as sample grouping are not available. We next examined the TCGA breast cancer (BRCA) dataset to see if the method can extract any new insights from the data.

### Real data analysis—TCGA breast cancer data

**Fig 5:**
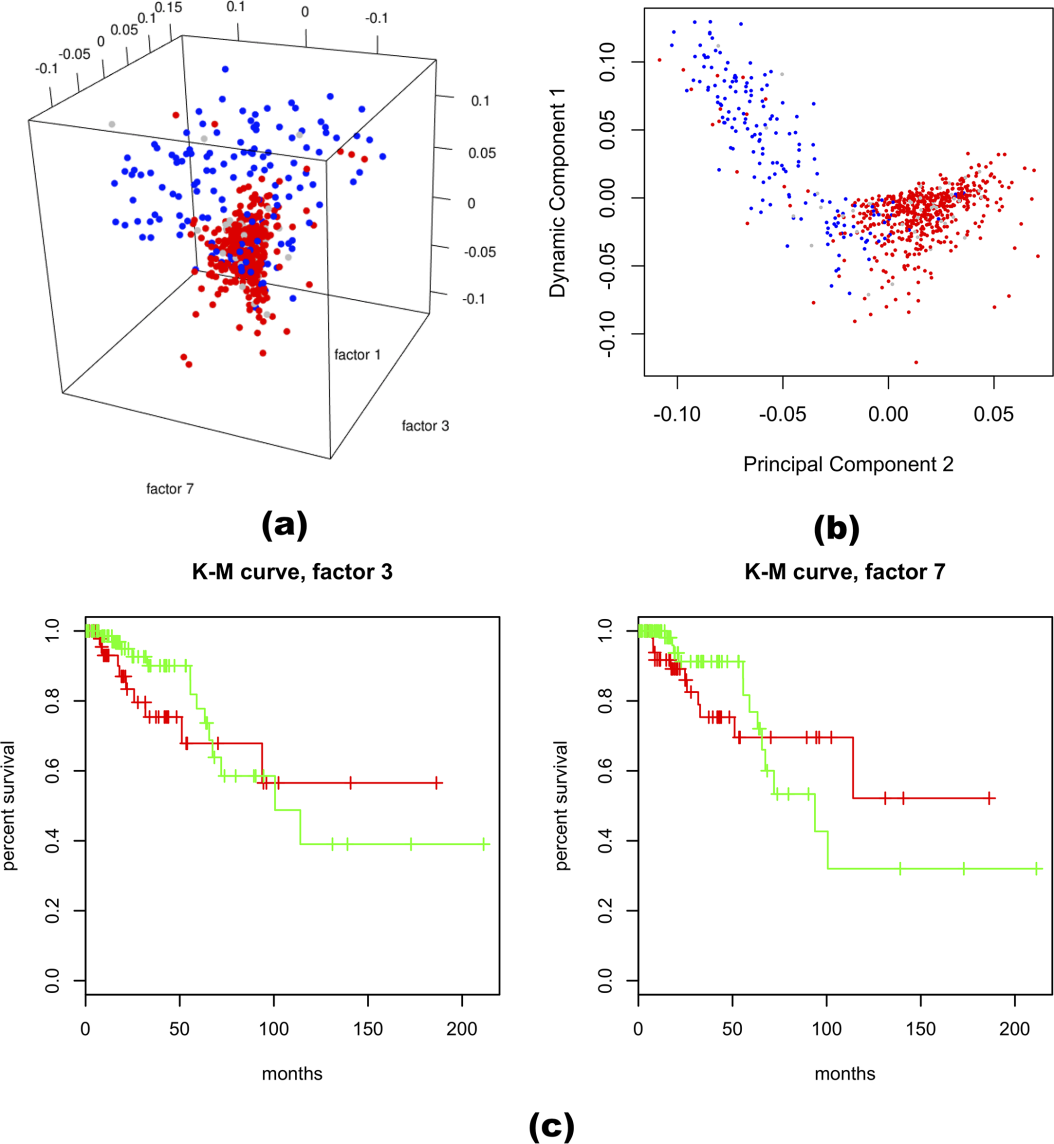
Results from the TCGA BRCA dataset. (a) Scatter plots of DC1, DC3, and DC7 scores. The points are colored based on the ER status of the subjects. DC1 separates ER+ and ER-, while DC3 and DC7 have a wide spread only for the ER- subjects. (b) DC1 captures similar information as the second principal component. (c) Kaplan–Meier curves of the ER-negative subjects, red: absolute factor score > 0.05.

The TCGA BRCA data contains the measurement of 20532 genes by deep sequencing in 762 subjects with breast cancer. After removing genes with >20% zero readings, 17728 genes remained in the study. Similar to the single cell RNA-seq data, DCA captured signals that were distinct from traditional methods. Here we focus our discussion on three of the DCs, as they are clearly linked to estrogen receptor (ER) status. Fig 5A shows the plot of the factor scores of these three DCs, each point corresponding to a subject. DC1 largely separated ER-positive and ER-negative samples, which agreed with the second principal component very well (Fig 5B). On the other hand, in the space spanned by DC3 and DC7, ER-positive samples were tightly clustered in the middle, while part of the ER-negative samples were spread widely (Fig 5A, S3 Fig). No PCs captured a similar structure in the data (S4 Fig).

Further analyses showed that among the ER-negative subjects, those with more extreme scores in either DC3 or DC7 showed a different survival characteristic than those in the center (Fig 5C). The subjects with more extreme scores tended to have a much higher chance of dying earlier, while in long follow-ups the remaining subjects tended to survive longer, albeit supported by relatively few data points.

**Fig 6:**
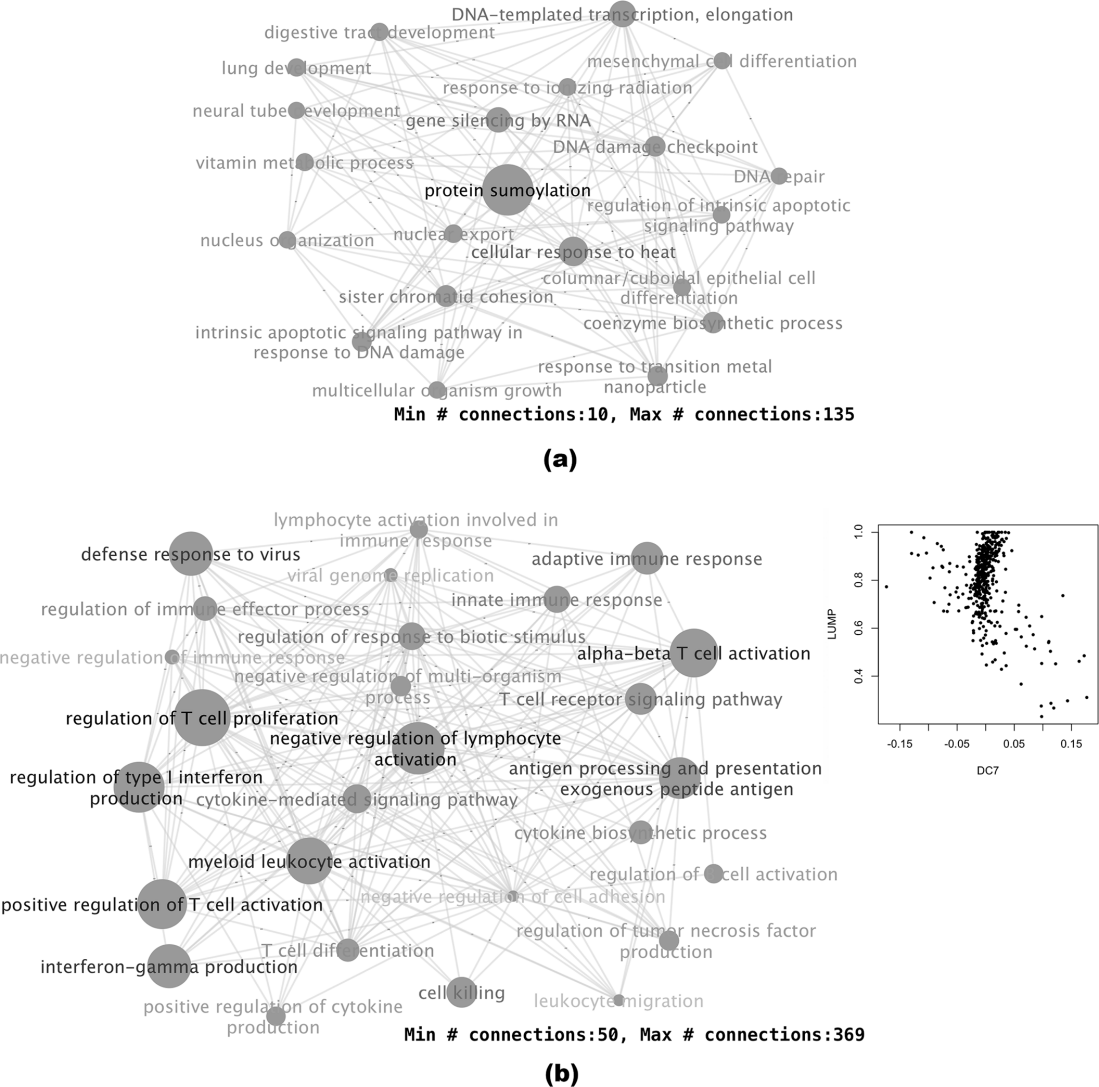
Biological process pairs with excessive dynamic correlations related to DCs 3 and 7. Gene pairs were selected using fdr threshold of 0.01. Biological process pairs were selected using a p-value threshold of 0.001 and fold-change of 3. For simplicity, only nodes with connections above a certain threshold are shown. Node sizes reflect the total number of connections of each node. (a) Biological process pairs associated with the 3^rd^ DC. (b) Biological process pairs associated with the 7^th^ DC. Inset: scatterplot of LUMP (leukocytes unmethylation for purity) vs DC7 score. The correlation coefficient is -0.35.

Functionally, the biological processes that showed excessive dynamic correlations conditioned on DC3 were centered around two main themes (Fig 6A). The first was protein sumoylation and stress response. Sumoylation is a post-translational modification that often occurs in response to cellular stress [20]. Many oncogenes and tumor suppressors are functionally related to sumoylation [21]. The second main theme was cell differentiation and tissue development that were related to several types of tissues, indicating a dysregulation in the cancer cells.

The biological processes associated with DC7 were mostly immune response processes (Fig 6B). Patterns of immune cell infiltration has been linked to the prognosis and treatment response of breast cancer [22]. The changed expression patterns of mostly immune-related genes in these samples were likely reflective of a certain immune cell infiltration pattern that had implications in prognosis [23]. We took the cell purity estimates based on LUMP (leukocytes unmethylation for purity) criterion, which was based on the average of 44 non-methylated immune-specific CpG sites and largely reflected immune cell infiltration [24]. As shown in the inset of Fig 6B, samples with high DC7 scores were those with low purities estimated by LUMP, while samples with low DC7 scores were a subset of those with higher purity scores. How these samples differ from the other high purity samples is an interesting point for future studies. Similarly, beside the three DCs that we discuss here, most of the other DCs showed clear functional implications, but require extra study beyond this manuscript to elucidate their biological meaning.

### Real data analysis—the yeast cell cycle microarray dataset

Thirdly, we analyzed the well-studied Spellman cell cycle gene expression data [25]. The dataset has been analyzed by many authors. The purpose of the analysis here was to demonstrate that DCA can extract information that was not reported before, yet clearly meaningful, and provided novel biological insights.

The cell cycle dataset includes four time-series experiments of the yeast cell cycle, each using a different method of synchronization. The total dimension is 6178 genes by 73 samples. Missing values were imputed by the K-nearest neighbor (KNN) method [26]. When all four time series datasets were combined into a single dataset, traditional methods such as PCA and SPCA [27] extracted signals that were consistent across the four time series (S5 Fig), but not signals that separated the four time series, except the first PC that captured an oscillating signal which was an artifact in the CDC15 time series data [28].

**Fig 7:**
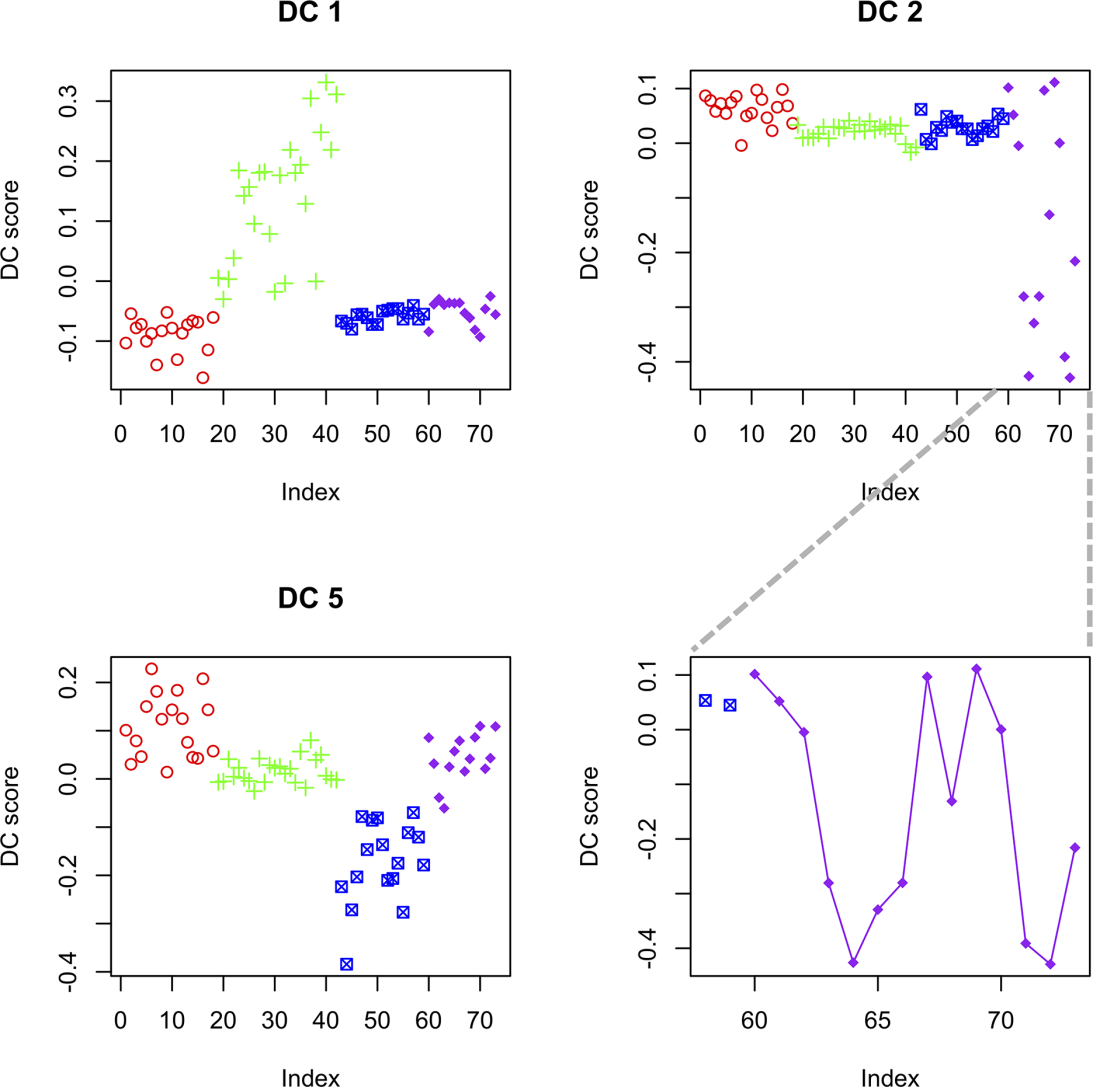
Some example Dynamic Components from the cell cycle data. Colors: the four cell cycle experiments. Red: alpha factor; green: CDC15; blue: CDC28; purple: elutriation.

Applying DCA to the combined cell cycle data yielded factors that were distinctly different. Most of the DCs clearly differentiated one of the four time series from the rest (S6 Fig). Here we focus our discussion on three of the factors. Fig 7 shows the plots of the scores of the three DCs, each point representing a sample. The first DC had high scores for samples from the CDC15 experiment only. It has been documented that an oscillating signal is present in the CDC15 data across many genes, causing an elevated level of correlation overall [28].

**Fig 8:**
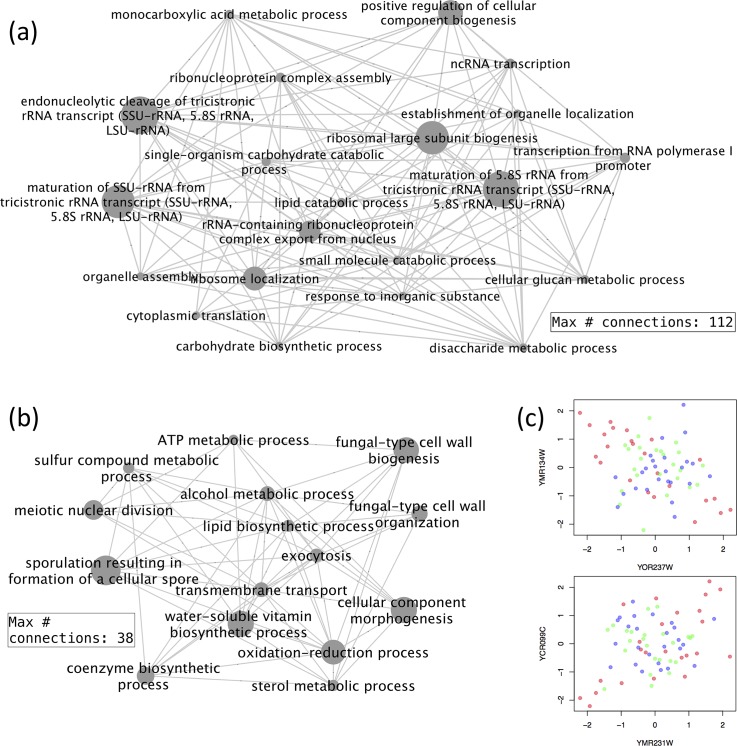
Biological process pairs with excessive dynamic correlations related to DCs 2 and 5. Gene pairs were selected using fdr threshold of 0.01. Biological process pairs were selected using a p-value threshold of 0.001 and fold-change of 2. For simplicity, only nodes with connections above a certain threshold are shown. Node sizes reflect the total number of connections of each node. (a) Biological process pairs associated with the DC2. (b) Biological process pairs associated with the DC5. (c) Example plots of gene pairs with LA relation with DC5. Red points: samples in the lower 33% of DC5 score; blue points: samples in the upper 33% of DC5 score.

The second DC only had extreme scores for some of the samples of the elutriation experiment. A closer examination revealed the DC showed a sine-wave pattern in the elutriation samples (Fig 7). An examination of the data revealed a strong dynamic correlation pattern between genes associated with this DC. Selecting biological processes pairs that had excessive dynamic correlation links between them, we found that the processes were focused on rRNA biogenesis and ribosome assembly (Fig 8A). Much more positive/negative correlations were shown between genes in these biological processes when the DC2 score is low, which corresponded to half of the samples in the elutriation experiment. While all the other three experiments were based on block-and-release cell cycle synchronization, the elutriation process separates synchronized cells based on their size, shape and mass [29]. The results here indicated that protein biosynthesis tended to be better synchronized in the elutriation samples compared to the other three experiments.

For the fifth DC, samples in the CDC28 experiment had lower scores, while the alpha factor samples had higher scores, with a smaller magnitude (Fig 7). This indicated that some gene pairs had a reverse correlation pattern between the two experiments, which was intriguing given both experiments used block-and-release to synchronize cells. Some recent studies have shed light on the metabolic behavior of the yeast cells under the alpha factor or CDC28 cell cycle arrest. Under the alpha factor treatment, the central metabolic fluxes are at a high level, and the cellular metabolism tend to be respiratory even when glucose is abundant [30]. The cell cycle CDK Cdc28 regulates both the cell division processes and metabolic processes. Under the CDC28 inhibition, the cells accumulate glycogen and trehalose to extremely high levels [31]. Given the different characteristics of the two cell cycle arrest mechanisms, it is understandable that after the release of cell cycle arrest, the cells proceed from very different metabolic situations, and metabolism will adapt to those situations. Functionally, we observed the highly connected biological processes mostly involve small molecule metabolism and transport (Fig 8B). Two typical pairs of genes are shown in Fig 8C, where clear dynamic correlation is observed.

Unlike traditional methods such as PCA and SPCA that identified commonalities, the DCA approach tended to find signals that differentiate the four underlying experiments, and revealed some important biological processes that behaved differently between the experiments. Given the existing knowledge on the dataset, these results validated that DCA extract new and meaningful information.

## Discussion

In this study, we developed a new method to detect major dynamic correlation signals from large gene expression matrices. A new measure of dynamic correlation between a pair of variables, the Liquid Association Coefficient (LAC), was developed to facilitate the discovery of the dynamic correlation signals. We used eigen value decomposition to find the DCs after the top gene pairs that were likely to be dynamically correlated were found by LAC scores, and a new ***H*** matrix was constructed from the gene pairs. Conceptually, other methods used to find latent factors, such as Independent Component Analysis (ICA) [32], Sparse Principal Component Analysis (SPCA) [27], Modular Latent Structure Analysis (MLSA) [33], or various clustering methods could also be applied to the ***H*** matrix.

In all three datasets, the latent factors found by DCA showed strong dynamic correlation relations with large numbers of gene pairs. Two of the datasets were RNAseq data, which tend to be skewed in distribution. As we discuss in the first subsection in METHODS, although normality is required of the ***z*** vector to make the LA score a valid estimate of the expected derivative of the correlation between the gene pair given *Z*, and our approach doesn’t have the normality constraint, it still finds ***z*** vectors that are correlated with the change of correlation of large numbers of gene pairs, hence recovering the dominant dynamic correlation signals without relying on the distribution assumption of Liquid Association. We examined if any gene could be good surrogates of these latent factors. In the intestinal epithelial data, the highest absolute value of Spearman correlation coefficient between any gene and any of the latent factors was 0.65 (S7 Fig). In the BRCA data and Spellman cell cycle data, the correlation coefficients were even lower, with maximum values of 0.43 and 0.55 respectively (S7 Fig). These results suggest that using genes as surrogate measurements is not as effective.

On the surface, our method bears some resemblance to kernel PCA with degree two polynomial kernel, in which the kernel is defined as *K*(***g***_*i*_,***g***_*j*_) = (⟨***g***_*i*_,***g***_*j*_⟩ + *κ*)^2^. However in fact the two methods are very different. In our method, when considering a pair of dynamically correlated genes, what’s involved in the downstream computation is the vector, (*g*_*i*1_*g*_*j*1_, *g*_*i*2_*g*_*j*2_, …, *g*_*iN*_*g*_*jN*_), instead of the inner product. We further analyzed all three real datasets using the kernel PCA approach. The results clearly showed that kernel PCA with degree two polynomial kernel could not discover the patterns found by our method (S8 Fig, S9 Fig, S10 Fig).

Overall, as a new unsupervised learning method for high dimensional data, DCA can extract new and useful information from the data. DCA complements existing dimension reduction methods to reveal more internal structure in the data that could lead to new biological discovery. The method is straight-forward, and the computation is efficient. The R package is available at https://cran.r-project.org/web/packages/DCA/index.html.

## Materials and methods

### Setup and the overall workflow

The data is in the form of an expression matrix, ***G***_*p*×*n*_, with *p* genes in the rows and *N* samples in the columns. We assume that all genes are normalized to have mean 0 and standard deviation 1. Thus the correlation between two genes represented by two row-vectors, ***g***_*i*_ and ***g***_*j*_, $r_{g_{i},g_{j}}$ is equal to *E*(***g***_*i*_***g***_*j*_).

Here we consider the situation where among the $\left(\begin{array}{c} p\\ 2 \end{array}\right)$ gene pairs, a small portion are dynamically correlated. Further, a small group of latent variables *Z*_*k*_, *k* = 1, …, *K*, govern the dynamic correlations of the majority of the dynamically correlated gene pairs. Which pair of genes are governed by which latent variable is unknown.

Ideally, we would like to estimate the latent variables, *Z*_*k*_, *k* = 1, …, *K*, as well as which gene pair is associated with which latent variable. However, in real datasets, the number of genes *p* is usually over 10^4^. Subsequently, the number of possible pairs is on the scale of 10^8^, making it nearly impossible to treat the gene pair–latent variable relation as missing value, e.g. using the Expectation-Maximization (EM) algorithm approach. Thus our goal is to develop a heuristic approach that involves dimension reduction to find good approximate solution efficiently.

Following the notations of Liquid Association [6], given a pair of genes ***g***_*i*_ and ***g***_*j*_, and a latent factor *Z*, let *g*(*Z*) = *E*(***g***_*i*_***g***_*j*_|*Z* = *z*) denote the conditional correlation of the two genes given *Z* = *z*. The LA score is defined as *LA*(***g***_*i*_***g***_*j*_|*Z*) = *Eg*′(*Z*), which is the expected change of correlation between ***g***_*i*_ and ***g***_*j*_ with respect to *Z*. As shown in [6], *LA*(***g***_*i*_***g***_*j*_|*Z*) = *E*(***g***_*i*_***g***_*j*_*Z*) and is estimated by $\frac{1}{N}\sum_{n=1}^{N}g_{i,n}g_{j,n}z_{n}$, if *Z* is standard normal. If a new vector ***h*** is generated, which is the entry-wise product of ***g***_*i*_ and ***g***_*j*_,

$$h_{n}=g_{i,n}g_{j,n},\;n=1,\ldots ,N,$$

then under the assumption of normality of *Z*, *LA*(***g***_*i*_***g***_*j*_|*Z*) is estimated by $\frac{1}{N}\sum_{n=1}^{N}h_{n}z_{n}$, which is proportional to the dot product between the corresponding vectors ***z*** and ***h***.

If the pair of genes ***g***_*i*_ and ***g***_*j*_ are governed by *Z*, then *LA*(***g***_*i*_***g***_*j*_|*Z*) = *Eg*′(*Z*) has a large absolute value, which means (***z*** ⋅ ***h***)^**2**^ is large. On the other hand, if the pair of genes ***g***_*i*_ and ***g***_*j*_ are not dynamically correlated with regard to *Z*, then (***z*** ⋅ ***h***)^**2**^ is small. Given the scaling of *Z* only linearly scales the LA scores, we can add the constraint that the vector ***z*** we are seeking is unit-length.

If we can somehow gather all gene pairs that are dynamically correlated, and construct a new matrix ***H***, each row of which being an ***h*** vector constructed from a dynamically correlated gene pair, then one good heuristic solution is to seek the ***z*** vectors sequentially, by applying eigen value decomposition to the matrix ***H***′***H***, which finds the solution to the following optimization problem:

$$z_{1}={\mathrm{argmax}}_{\|z\|=1}\sum_{m}{\left(z∙h_{m}\right)}^{2},$$

$$z_{k}={\mathrm{argmax}}_{\|z\|=1}\sum_{m}{\left(z∙h_{m}\right)}^{2},\;s.t.\;z^{\prime }z_{l}=0,\;l=1,\ldots ,k-1,$$

where *m* indexes all the ***h*** vectors. The more gene pairs a latent *Z* variable regulates, the larger the sum of squared projection length. This way, the top eigen vectors of the ***H***′***H*** matrix capture the major signals that regulate the dynamic correlation of the majority of the dynamically correlated gene pairs. We name these vectors Dynamic Components (DCs). They are each of length *N*, which is the number of samples.

We note that for the quantity $\frac{1}{N}\sum_{n=1}^{N}h_{n}z_{n}$ to be a valid estimate of *LA*(***g***_*i*_***g***_*j*_|*Z*), i.e. the expected derivative of the correlation between ***g***_*i*_ and ***g***_*j*_ with respect to *Z*, the normality assumption of *Z* needs to hold. However, this is not guaranteed in the above estimation procedure. On the other hand, the above procedure seeks ***z*** vectors on which large numbers of ***h*** vectors have big projections, i.e. projection directions that are correlated with large numbers of $r_{g_{i},g_{j}}$. Thus even without the normality assumption, such ***z*** vectors are highly correlated with the change of correlation between many dynamically correlated gene pairs, meaning they are good estimates of the latent dynamic correlation signal. At the same time, with many data types, such as RNA-seq or LC/MS metabolomics data, the data itself is highly skewed. There is no reason to believe the underlying latent factors that govern dynamic correlation are normal. Thus loosening the assumption may be beneficial in the discovery of the true latent factors.

To apply this approach, the key is to find the dynamically correlated gene pairs from the ~10^8^ possible pairs. We find gene pairs that are dynamically correlated by ranking all pairs of genes using a newly developed metric, Liquid Association Coefficient (LAC), which is described in the next subsection. We should note that we cannot guarantee all dynamically correlated pairs are found, nor there are no noise pairs among the selected pairs. However, with the dimension reduction approach being applied, missing some pairs or including some noise pairs, as long as they do not account for too large a proportion in the ***H*** matrix, the main latent factors can still be recovered.

### Selecting gene pairs that are likely to be dynamically correlated

For the purpose of selecting informative gene pairs, we define a measure for dynamic correlation between a pair of genes, the Liquid Association Coefficient (LAC), which can take two forms. The first is the correlation coefficient of the squared values of the two genes, minus the correlation coefficient of the original values squared.

$$\zeta_{i,j}=r(g_{i}^{2},g_{j}^{2})-r^{2}(g_{i},g_{j}),$$

where *r*() is the Pearson’s correlation coefficient. It has been shown that when both *g*_*i*_ and *g*_*j*_ follow the bivariate normal distribution with mean $\left(\begin{array}{c} 0\\ 0 \end{array}\right)$, and variance-covariance matrix $\left(\begin{array}{cc} 1 & \rho \\ \rho & 1 \end{array}\right)$, the above quantity converges to zero no matter what value *ρ* takes.

Alternatively, to reduce the impact of more extreme values, we can use the correlation coefficient of the absolute values of the two genes minus the absolute value of the correlation coefficient:

$$\zeta_{i,j}=r\left(|g_{i}|,|g_{j}|\right)-\left|r(g_{i},g_{j})\right|.$$

We compute the matrix of *LAC* values for all pairs of genes. Notice the computational cost is on the same scale as computing the pairwise correlation matrix. We then select the (*i*,*j*) pairs whose *LAC* values are above a certain percentile of all the values in the matrix. In this study, we use top 2.5% or 10^6^ pairs, whichever is smaller.

### Finding DCs and their associated gene pairs

After selecting the top (*i*,*j*) pairs, we construct the ***H*** matrix, in which each row is constructed from a selected pair of genes. For example, if ***g***_***i***_ and ***g***_***j***_ are selected as a pair of informative genes, then the corresponding row of the new matrix is (*g*_*i*1_*g*_*j*1_, *g*_*i*2_*g*_*j*2_, …, *g*_*iN*_*g*_*jN*_). We then find a sequence of latent factors using eigenvalue decomposition on the matrix ***H***′***H***.

In order to improve the interpretability of the resulting factors, further factor rotations can be conducted to better align the DCs with groups of ***h*** vectors (gene pairs). In this study, we used the varimax rotation, which rotates the latent factors in the subspace they span, and seeks to maximize the sum of the variances of the squared loadings of the ***h*** vectors on the latent factors [34].

To find the gene pairs associated with each of the DCs, we first calculate the LAC coefficients for all pairs of genes, and select gene pairs with *LAC* coefficients belonging to a top percentile (20% in this study). For each selected ***(g***_***i***_**, *g***_***j***_***)*** pair, we construct the ***h*** vector,

$$h_{n}=g_{i,n}g_{j,n},\;n=1,\ldots ,N.$$

For a ***z*** vector, we calculate its dot product with all the ***h*** vectors that are constructed from the selected pairs,

$$\gamma_{m}=\sum_{n=1}^{N}z_{n}h_{n}^{\left(m\right)},\;n=1,\ldots ,N,m=1,\ldots ,M$$

where m indexes the ***h*** vectors, and *M* is the total number of gene pairs used. According to the Central Limit Theorem, the dot products approximately follow a normal distribution when the ***z*** vector is independent of an ***h*** vector, i.e. a ***(g***_***i***_**, *g***_***j***_***)*** pair. As we now consider a large number of gene pairs (20% of all possible pairs, on 10^7^ scale), we can safely assume the majority of the gene pairs don’t have a dynamic correlation with regard to a given ***z*** vector, while a small portion of the dot products follow another distribution as the corresponding pairs are dynamically correlated with regard to ***z***. Thus together, ${\left\{\gamma_{m}\right\}}_{m=1}^{M}$ follow a mixture distribution. This is very similar to the considerations in the local false discovery rate (lfdr) literature. We consider the density of ${\left\{\gamma_{m}\right\}}_{m=1}^{M}$ as a mixture with two components:

$$f(\gamma )=\pi_{0}f_{0}(\gamma )+(1-\pi_{0})f_{1}(\gamma )$$

where *f*() is the mixture density for the observed *γ* statistic, *f*_0_() and *f*_1_() are the respective densities of the null (unassociated with ***z***) and non-null (associated with ***z***) gene pairs, and *π*_0_ is the proportion of the true null gene pairs. Then the posterior probability that a gene pair belongs to the null distribution is $\pi_{0}\frac{f_{0}(\gamma )}{f(\gamma )}$, at any value of the *γ* statistic.

Given the similarity of the setup, we can simply borrow from the mature local false discovery rate (lfdr) methods. For every ***z*** vector, we generate the collection of *γ* statistics ${\left\{\gamma_{m}\right\}}_{m=1}^{M}$, with each element corresponding to a gene pair. We then apply the existing local false discovery rate (lfdr) method to calculate the posterior probability that a gene pair belongs to the null distribution [35], and threshold the lfdr values to select gene pairs that are dynamically correlated given the latent factor.

### Finding biological processes associated with a latent factor

For functional interpretation, we use gene ontology (GO) biological processes. We first select a set of representative GO biological process terms that are of reasonable size and relatively small overlaps, following an existing procedure that considers both the ontology structure and the number of genes assigned to each term [36]. For the mouse data, we select 428 biological processes with 100~1000 assigned genes each, covering 15161 genes in total. For the human data, we select 423 biological processes with 100~1000 assigned genes each, covering 14414 genes in total. For the yeast data, we select 172 biological processes with 50~1000 assigned genes each, covering 5334 genes in total. From the gene pairs associated with each latent factor, we conduct two types of analyses:

#### Within-process dynamic correlation

For each biological process, we count the occurrence of gene pairs in which both genes fall into the process. We also calculate the expected number of such gene pairs if all the gene pairs were randomly drawn. We calculate the fold-change by taking the ratio of observed count *v*.*s*. the expected count, and p-value using the binomial distribution.

#### Between-process dynamic correlation

For each pair of selected biological processes, we first remove their overlapping genes. We then count the occurrence of gene pairs in which the two genes fall into the two processes respectively, and calculate the expected number of such gene pairs if all the genes were randomly drawn. We then calculate the fold-change by taking the ratio, and p-value using the binomial distribution. After thresholding the fold change and p-value to select pairs of processes, we visualize the resulting network using Cytoscape [37].

## Supporting information

S1 Fig Simulation result of setup 2. (a) The marginal distributions of gene expression levels were normal. (b) The marginal distributions of gene expression levels mimicked real RNA-seq data. Row sub-plots: number of genes in each module; Columns subplots: the number of modules; Line color: sample size; line type: method used for latent factor recovery. Given the heavy computational cost, the “LA screening (upper limit)” results were obtained by directly selecting the genes that have the highest absolute correlation with the hidden factors, meaning the values plotted are the best possible, but may not be attainable in actual computation.
(TIF)

S2 Fig Simulation result of setup 3. (a) The marginal distributions of gene expression levels were normal. (b) The marginal distributions of gene expression levels mimicked real RNA-seq data. Row sub-plots: number of genes in each module; Columns subplots: the number of modules; Line color: sample size; line type: method used for latent factor recovery. Given the heavy computational cost, the “LA screening (upper limit)” results were obtained by directly selecting the genes that have the highest absolute correlation with the hidden factors, meaning the values plotted are the best possible, but may not be attainable in actual computation.
(TIF)

S3 Fig Pairwise scatter plots of DC factors 1, 3 and 7. Red points: ER-positive; Blue points: ER-negative; Grey points: unknown status.
(TIF)

S4 Fig Pairwise scatter plots of the first 8 principal components of the BRCA data. Red points: ER-positive; Blue points: ER-negative; Grey points: unknown status.
(TIF)

S5 Fig Principal components of the cell cycle data. (TIF)

S6 Fig Dynamic Components (DCs) of the cell cycle data. (TIF)

S7 Fig The maximum absolute Spearman correlation between each latent factor and any gene in the dataset. (a) Intestinal epithelial dataset. (b) TCGA BRCA dataset. (c) Spellman cell cycle dataset.
(TIF)

S8 Fig Kernel PCA results from the mouse intestine single cell RNAseq data. Degree 2 polynomial kernel was used to generate the results.
(TIF)

S9 Fig Kernel PCA results from the TCGA BRCA data. Degree 2 polynomial kernel was used to generate the results.
(TIF)

S10 Fig Kernel PCA results from the Yeast cell cycle data. Degree 2 polynomial kernel was used to generate the results.
(TIF)
