# Supplementary figures and images for: A new dynamic correlation algorithm reveals novel functional aspects in single cell and bulk RNA-seq data
Source: PLoS Comput Biol. 2018 Aug 6;14(8):e1006391. doi: 10.1371/journal.pcbi.1006391 (PMC6095616; doi:10.1371/journal.pcbi.1006391)

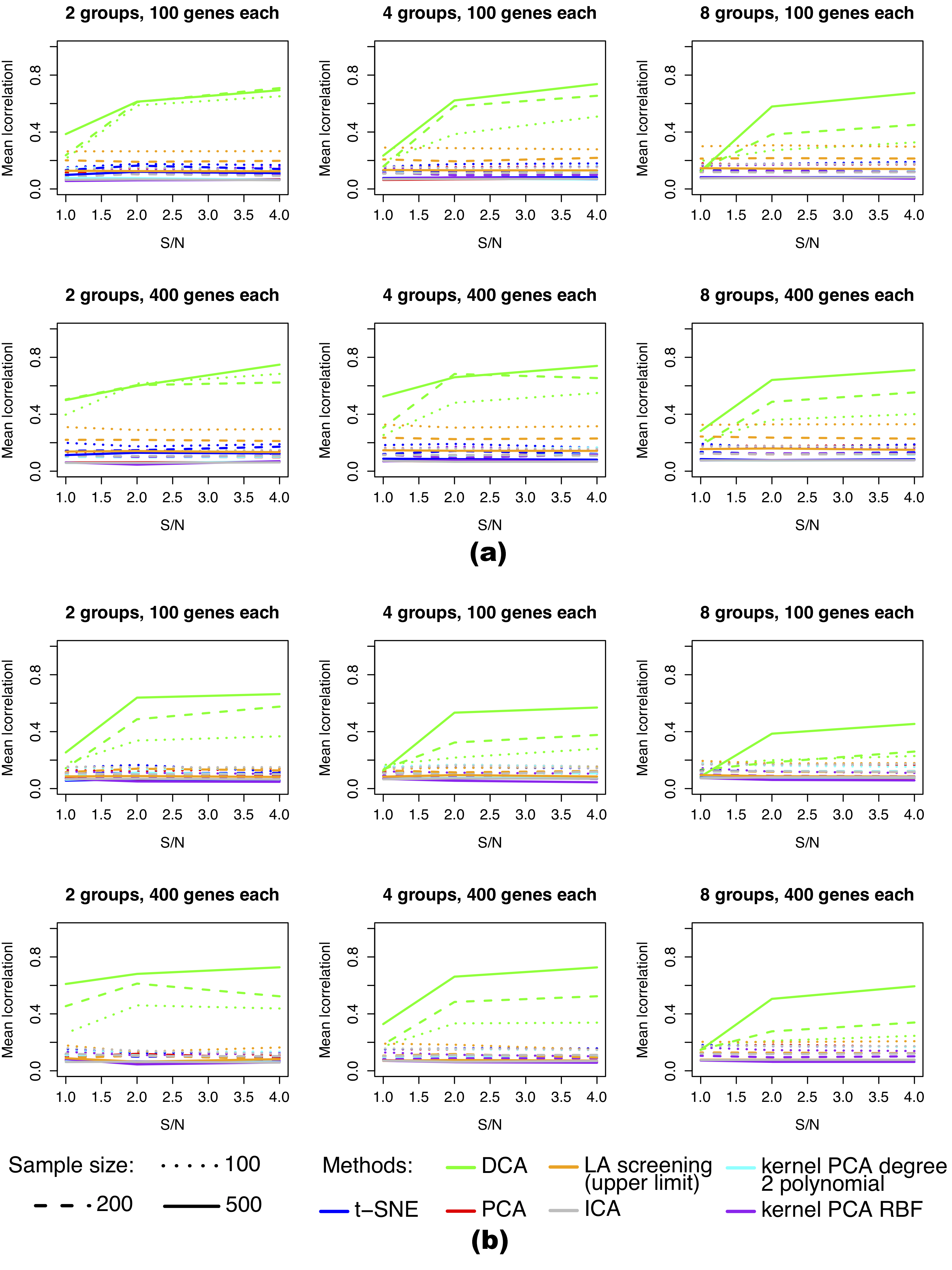

Supplement: S1 Fig — (a) The marginal distributions of gene expression levels were normal. (b) The marginal distributions of gene expression levels mimicked real RNA-seq data. Row sub-plots: number of genes in each module; Columns subplots: the number of modules; Line color: sample size; line type: method used for latent factor recovery. Given the heavy computational cost, the “LA screening (upper limit)” results were obtained by directly selecting the genes that have the highest absolute correlation with the hidden factors, meaning the values plotted are the best possible, but may not be attainable in actual computation. (TIF) [file pcbi.1006391.s001.tif]

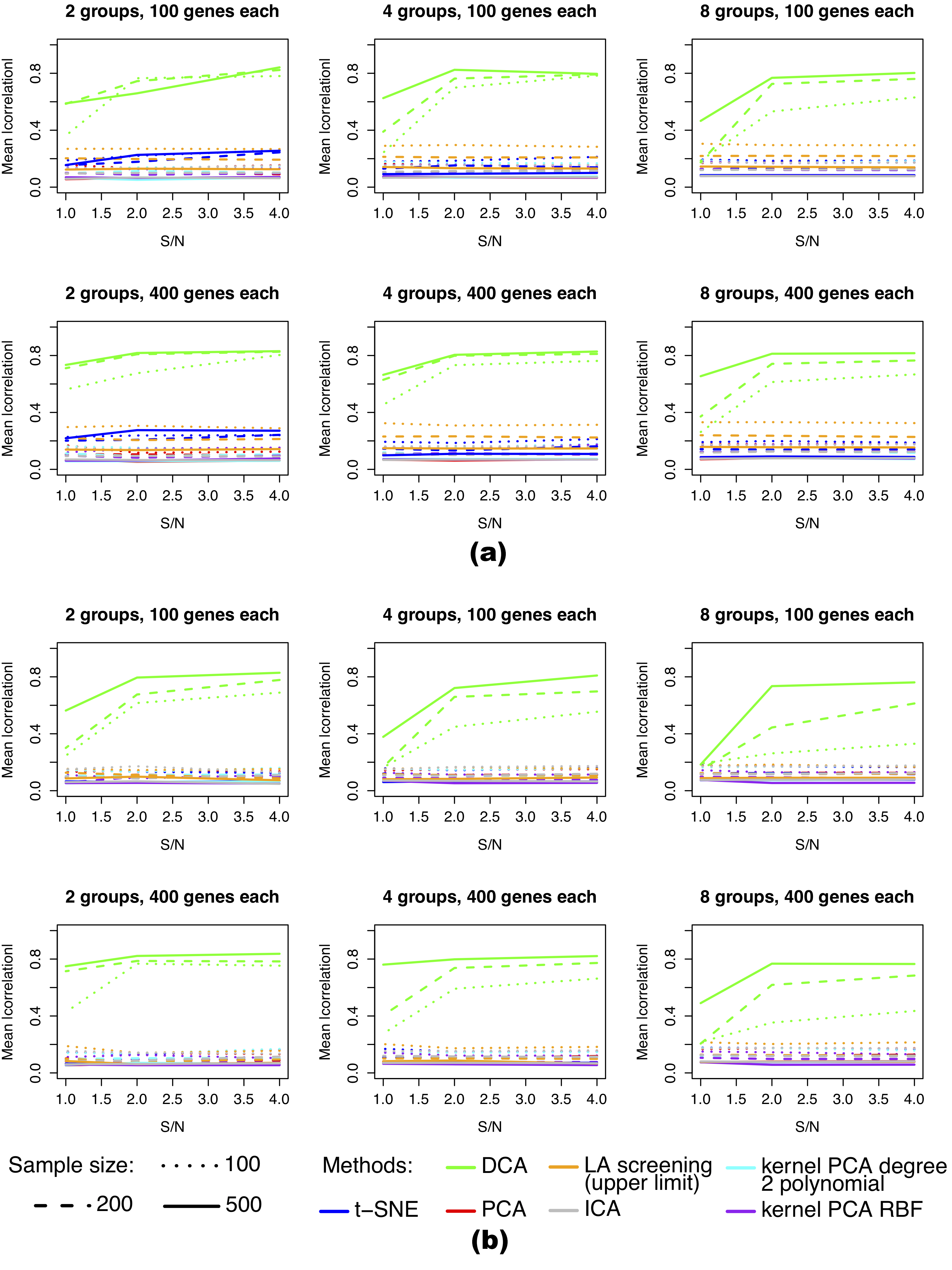

Supplement: S2 Fig — (a) The marginal distributions of gene expression levels were normal. (b) The marginal distributions of gene expression levels mimicked real RNA-seq data. Row sub-plots: number of genes in each module; Columns subplots: the number of modules; Line color: sample size; line type: method used for latent factor recovery. Given the heavy computational cost, the “LA screening (upper limit)” results were obtained by directly selecting the genes that have the highest absolute correlation with the hidden factors, meaning the values plotted are the best possible, but may not be attainable in actual computation. (TIF) [file pcbi.1006391.s002.tif]

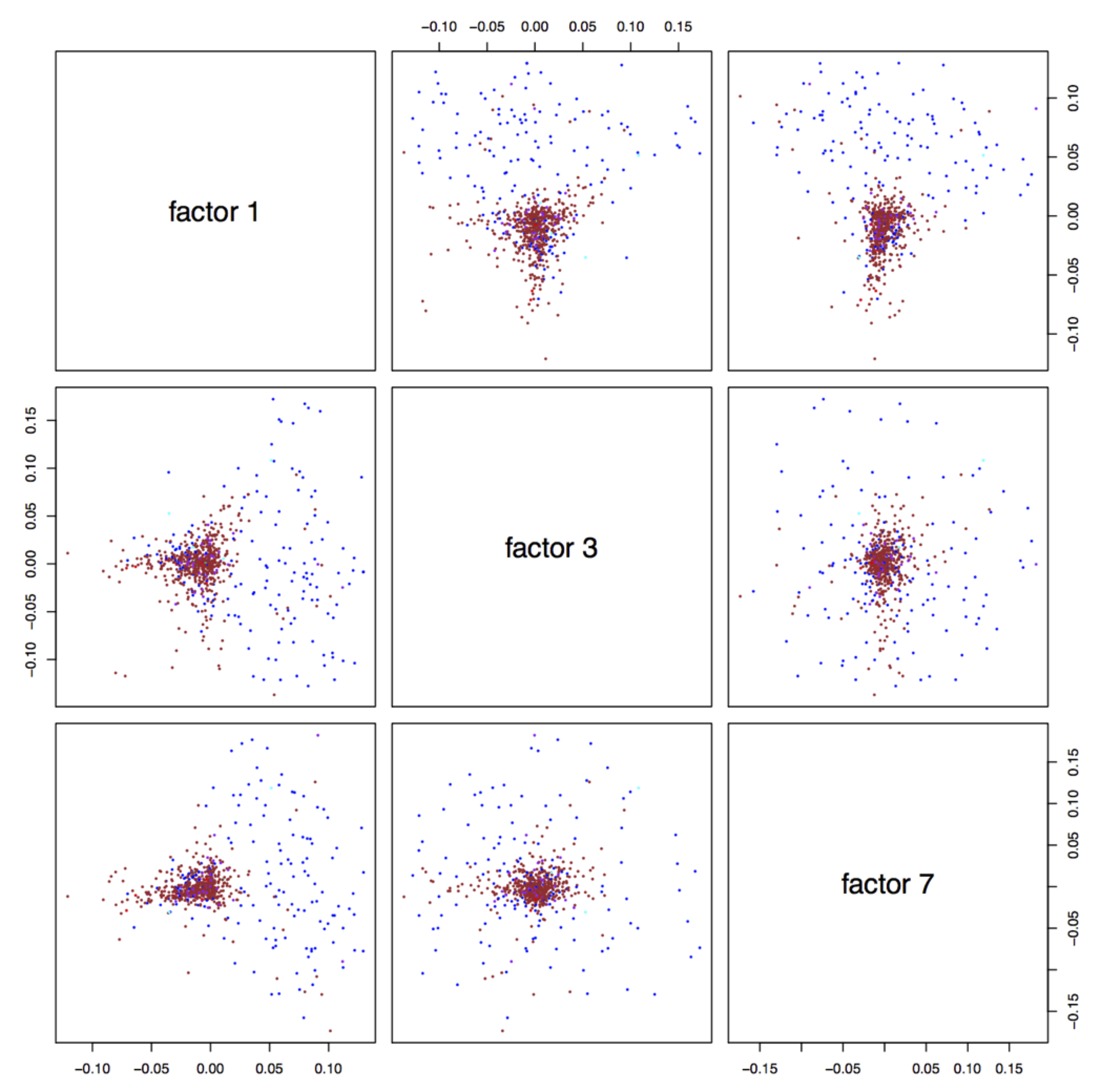

Supplement: S3 Fig — Red points: ER-positive; Blue points: ER-negative; Grey points: unknown status. (TIF) [file pcbi.1006391.s003.tif]

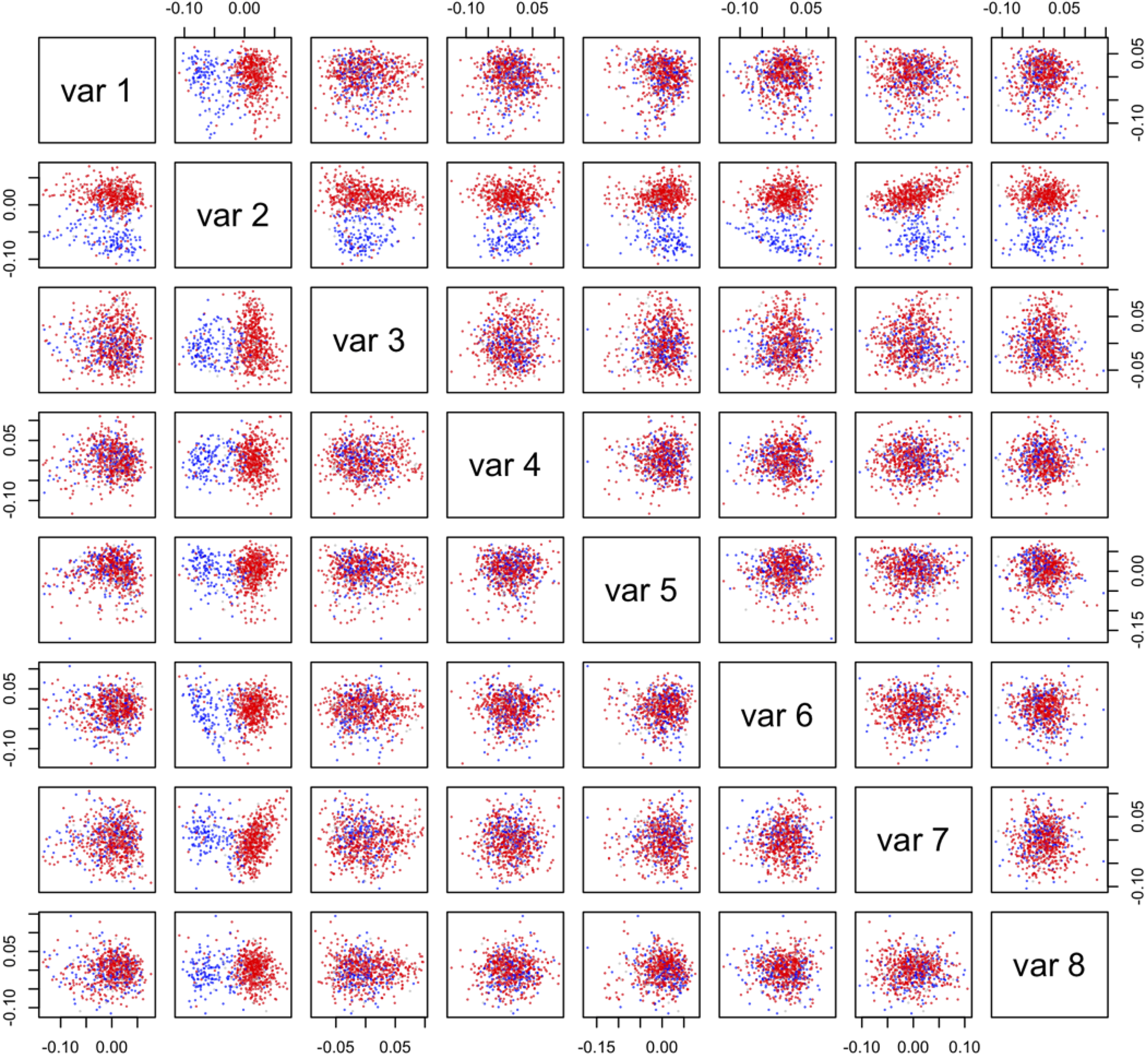

Supplement: S4 Fig — Red points: ER-positive; Blue points: ER-negative; Grey points: unknown status. (TIF) [file pcbi.1006391.s004.tif]

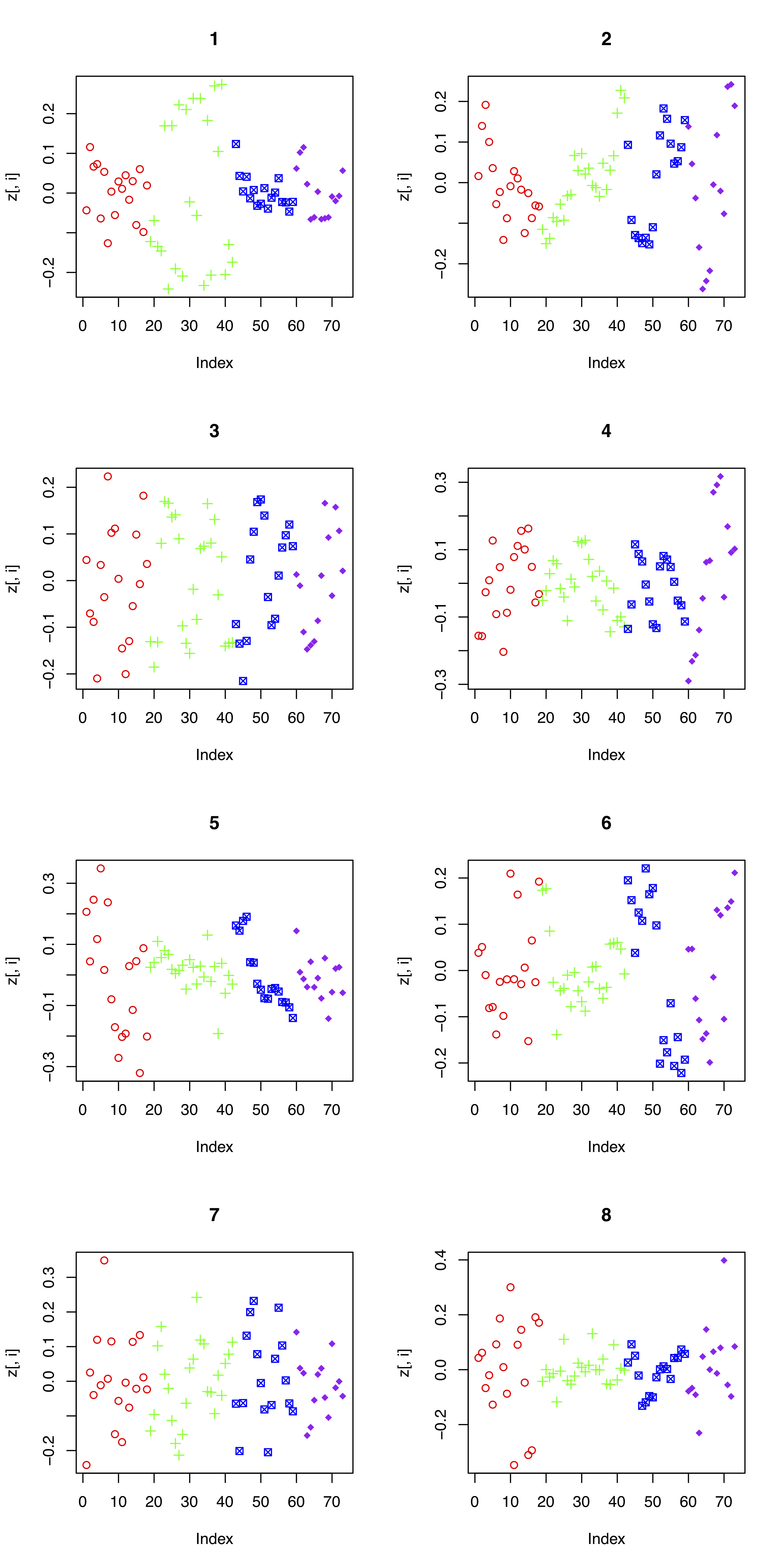

Supplement: S5 Fig — (TIF) [file pcbi.1006391.s005.tif]

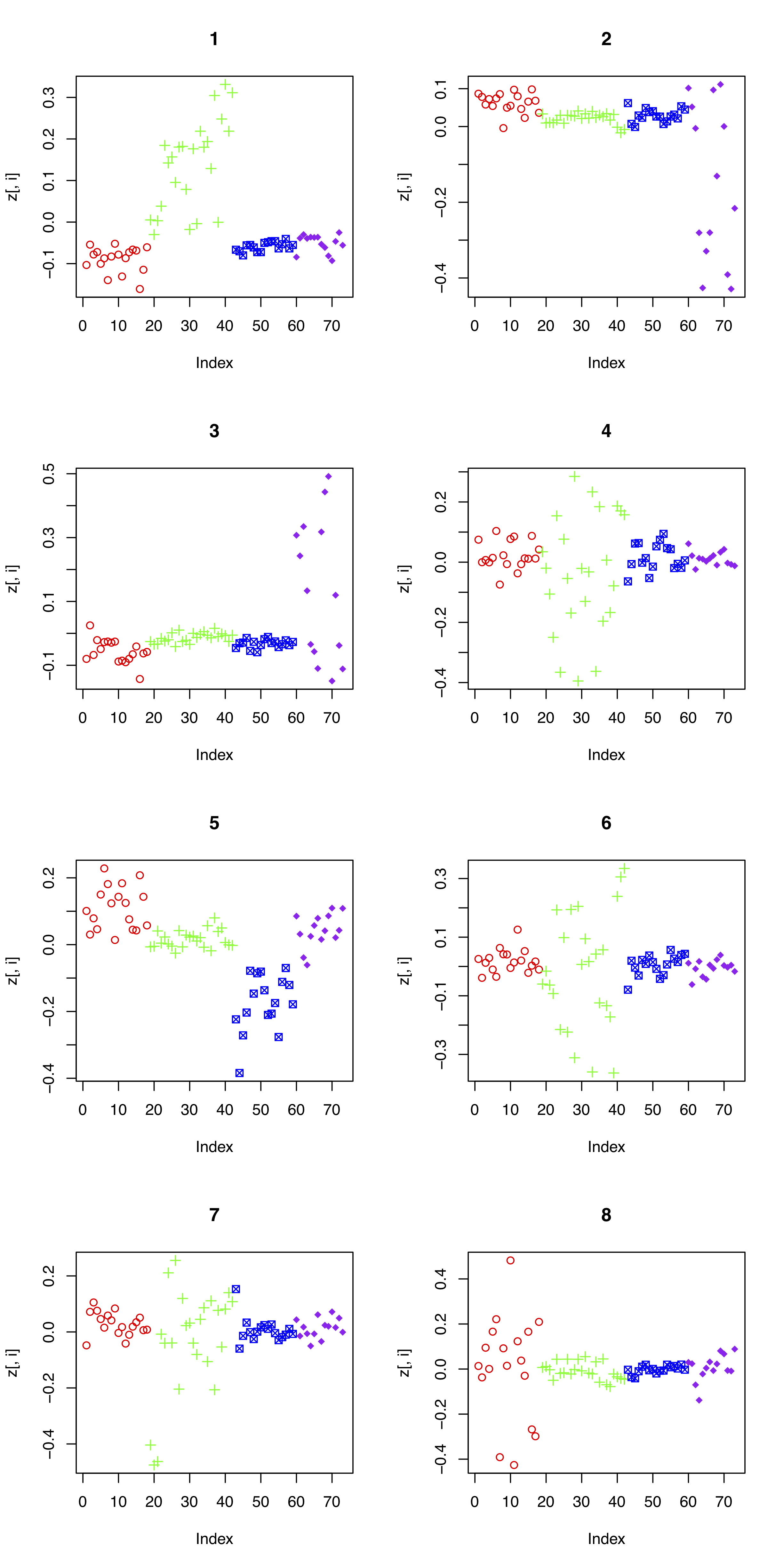

Supplement: S6 Fig — (TIF) [file pcbi.1006391.s006.tif]

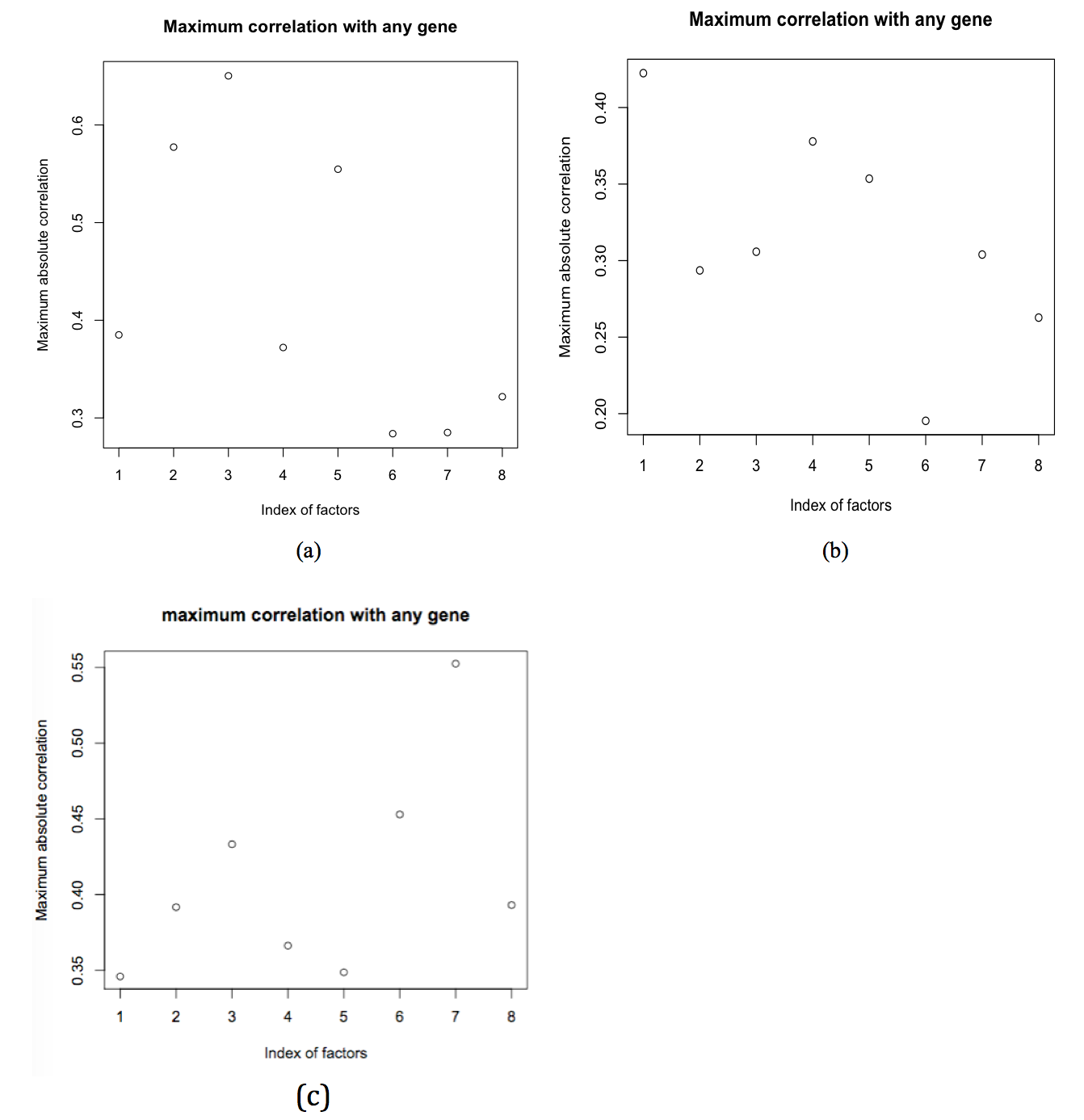

Supplement: S7 Fig — (a) Intestinal epithelial dataset. (b) TCGA BRCA dataset. (c) Spellman cell cycle dataset. (TIF) [file pcbi.1006391.s007.tif]

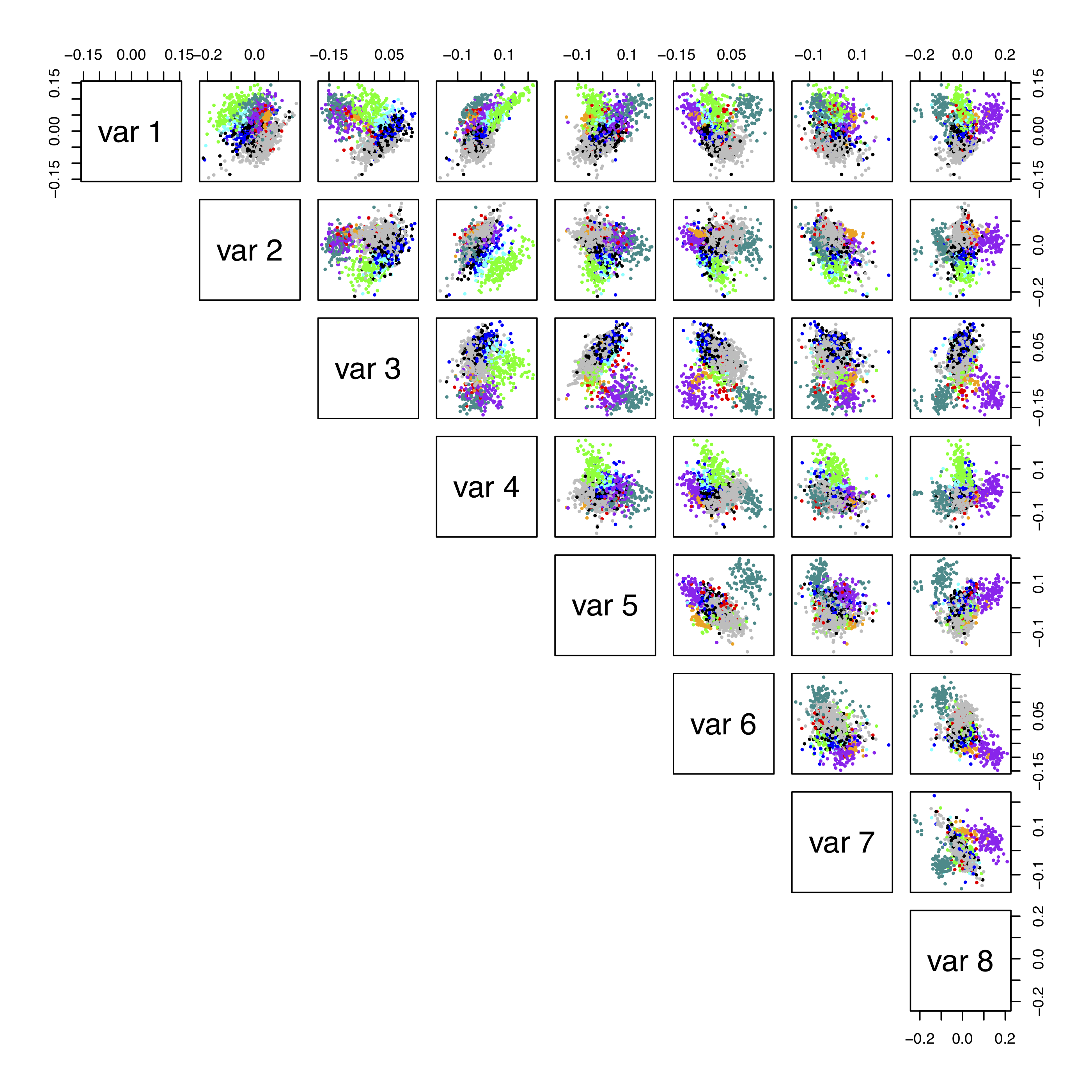

Supplement: S8 Fig — Degree 2 polynomial kernel was used to generate the results. (TIF) [file pcbi.1006391.s008.tif]

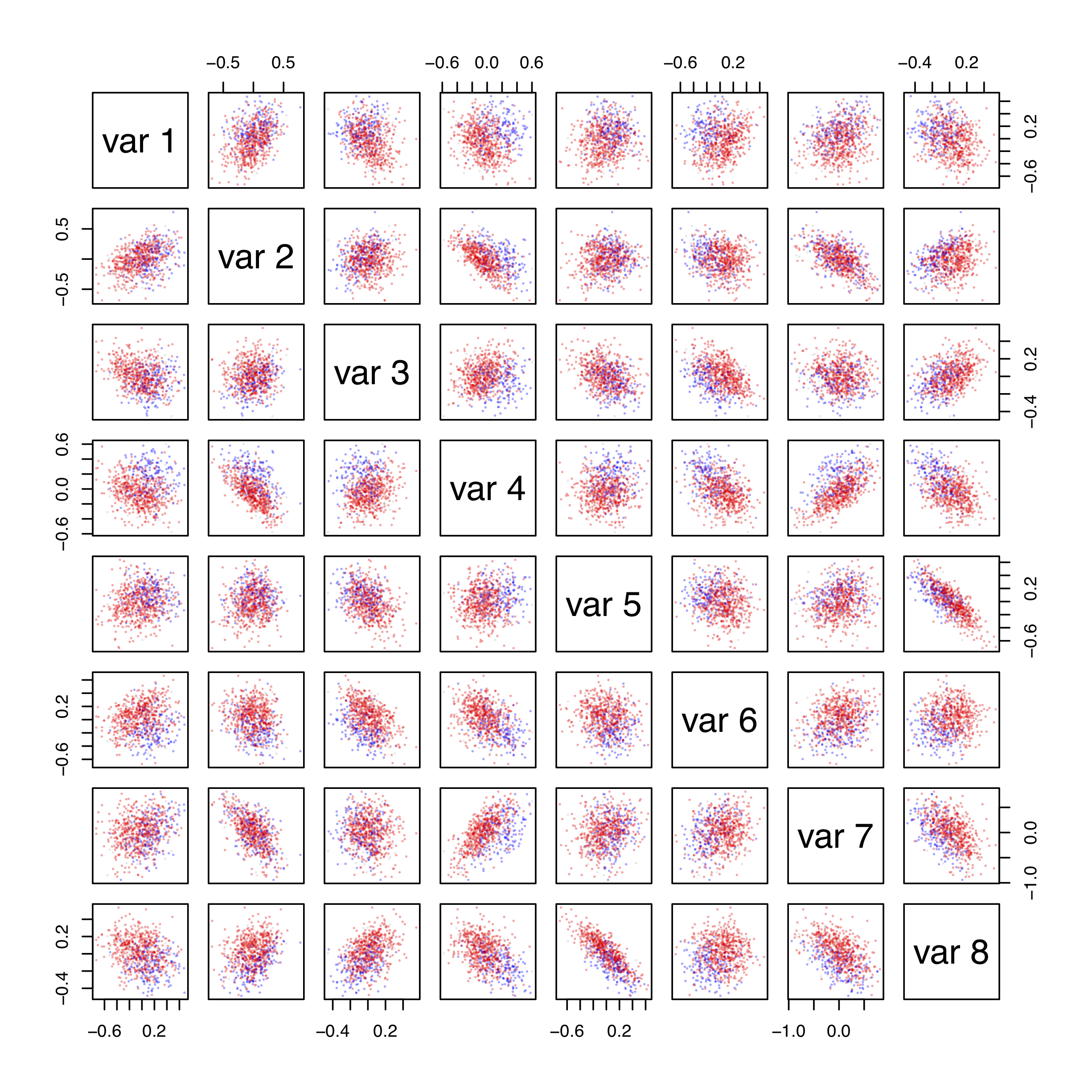

Supplement: S9 Fig — Degree 2 polynomial kernel was used to generate the results. (TIF) [file pcbi.1006391.s009.tif]

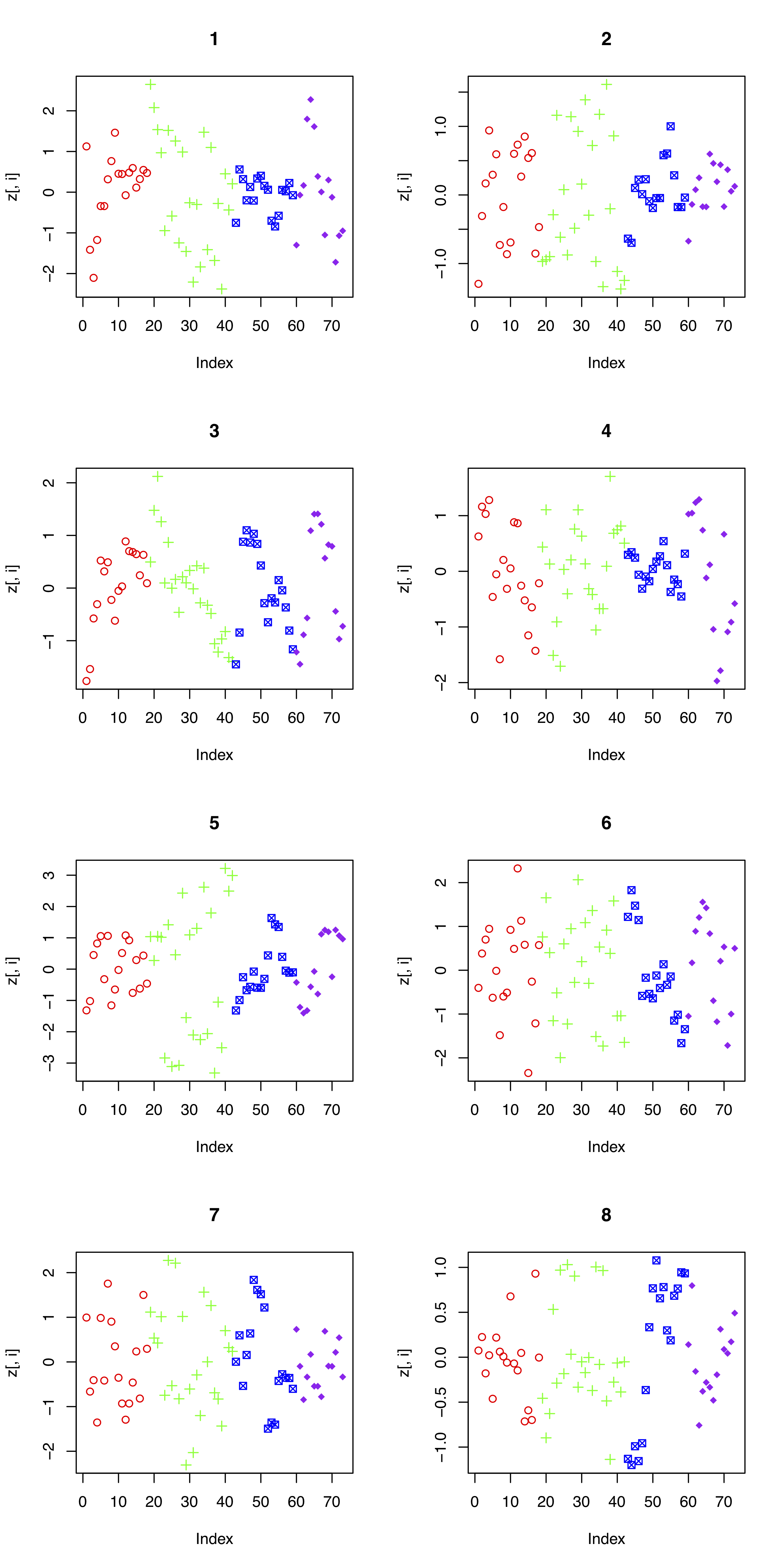

Supplement: S10 Fig — Degree 2 polynomial kernel was used to generate the results. (TIF) [file pcbi.1006391.s010.tif]
